# Single-cell transcriptomics reveals immune suppression and cell states predictive of patient outcomes in rhabdomyosarcoma

**DOI:** 10.1038/s41467-023-38886-8

**Published:** 2023-05-27

**Authors:** Jeff DeMartino, Michael T. Meister, Lindy L. Visser, Mariël Brok, Marian J. A. Groot Koerkamp, Amber K. L. Wezenaar, Laura S. Hiemcke-Jiwa, Terezinha de Souza, Johannes H. M. Merks, Anne C. Rios, Frank C. P. Holstege, Thanasis Margaritis, Jarno Drost

**Affiliations:** 1 Princess Máxima Center for Pediatric Oncology, Heidelberglaan 25, 3584 CS Utrecht, The Netherlands; 2 Oncode Institute, Heidelberglaan 25, 3584 CS Utrecht, The Netherlands; 3 Department of Pathology, University Medical Center Utrecht, Utrecht, The Netherlands; 4 Center for Molecular Medicine, UMC Utrecht and Utrecht University, Universiteitsweg 100, 3584 CG Utrecht, the Netherlands

**Keywords:** Cancer genomics, Paediatric cancer, Cancer microenvironment

## Abstract

Paediatric rhabdomyosarcoma (RMS) is a soft tissue malignancy of mesenchymal origin that is thought to arise as a consequence of derailed myogenic differentiation. Despite intensive treatment regimens, the prognosis for high-risk patients remains dismal. The cellular differentiation states underlying RMS and how these relate to patient outcomes remain largely elusive. Here, we use single-cell mRNA sequencing to generate a transcriptomic atlas of RMS. Analysis of the RMS tumour niche reveals evidence of an immunosuppressive microenvironment. We also identify a putative interaction between NECTIN3 and TIGIT, specific to the more aggressive fusion-positive (FP) RMS subtype, as a potential cause of tumour-induced T-cell dysfunction. In malignant RMS cells, we define transcriptional programs reflective of normal myogenic differentiation and show that these cellular differentiation states are predictive of patient outcomes in both FP RMS and the less aggressive fusion-negative subtype. Our study reveals the potential of therapies targeting the immune microenvironment of RMS and suggests that assessing tumour differentiation states may enable a more refined risk stratification.

## Abstract (web summary)

The cellular differentiation states of paediatric rhabdomyosarcoma (RMS) remain to be explored. Here, single-cell RNA sequencing analysis of RMS tumours reveals an immunosuppressive microenvironment and distinct transcriptional programs predictive of patient outcomes.

## Introduction

Rhabdomyosarcoma (RMS) is the most commonly diagnosed soft tissue sarcoma (STS) in children and adolescents, accounting for ~3.5% of all paediatric malignancies^1^. Several characteristics, including expression of the myogenic regulatory transcription factors *MYOD1* and *MYOG*^2^ and the presence of rhabdomyoblasts^3^ (cells reminiscent of terminally differentiating myocytes), point to RMS being the result of impaired skeletal muscle myogenesis. However, the disease may also arise at body sites devoid of skeletal muscle, and RMS models of non-myogenic origin have been described^4^. Despite intense, multimodal treatment strategies, outcomes remain dismal for patients with high-risk or metastatic disease, the latter of which exhibits a long-term overall survival rate (OS) of ~30%^5^. This emphasises the need to improve our understanding of RMS tumour biology to enable the development of novel therapeutic approaches.

Historically, RMS has been divided into two main subtypes, alveolar and embryonal, based on the histological features of tumours^6^. However, recent work has shown that the molecular classification as either fusion-positive (FP) or fusion-negative (FN) is a more powerful prognostic indicator^7,8^. FP RMS is characterised by recurrent chromosomal translocations resulting in the expression of a chimeric fusion protein containing the DNA binding domains of either PAX3 or PAX7, both key transcriptional regulators of normal myogenesis^9^, coupled to a strong transactivation domain, most often that of FOXO1^10,11^. The genetic lesions driving FN RMS, on the other hand, are diverse and may include mutations in signal transduction pathways (especially RAS and PI3K), cell cycle regulators and the P53 pathway, among others^12^. Notably, FP RMS carries a significantly worse prognosis than FN RMS and is more often metastatic at diagnosis^8^.

In addition to the inter-tumoral genetic heterogeneity characteristic of RMS, it has been recognised that there exists a degree of heterogeneity within tumours, as exemplified by the diversity in cellular morphology^13^ and variation in immunohistochemical staining for myogenic markers^14^. However, the characteristics and clinical implications of this heterogeneity remain unclear. In addition, the composition of the tumour microenvironment (TME) and the interplay between malignant cells and the TME have not been comprehensively profiled.

Here, we compile a single-cell transcriptomic atlas comprising both FN and FP RMS and find distinct differences in cellular composition and differentiation states between and within subtypes that relate to clinical outcomes and suggest potential immunotherapeutic interventions.

## Results

### A single-cell atlas of paediatric RMS tumours

**Fig. 1:**
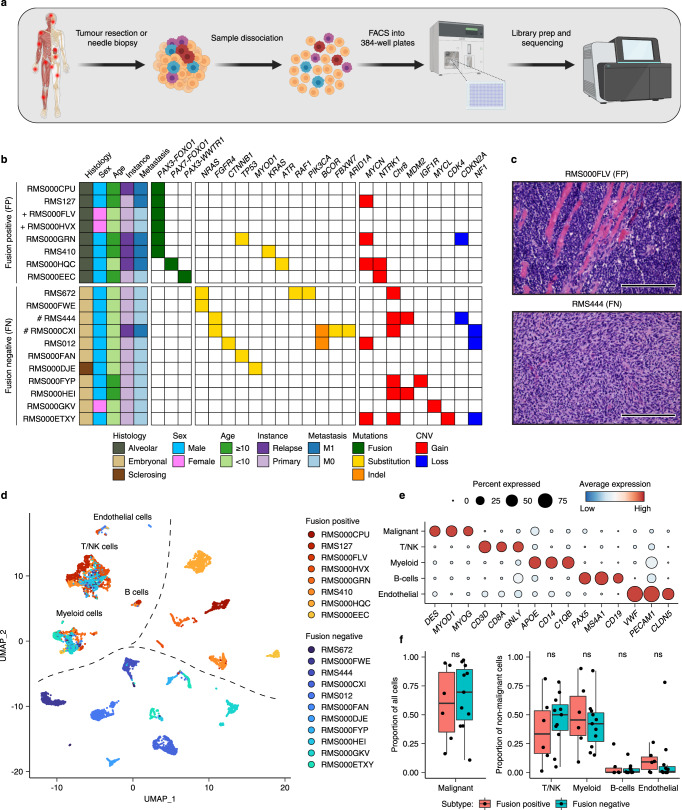
Single-cell transcriptomic atlas of RMS tumours. **a** Schematic representation of the sample processing workflow used to generate scRNA-seq data from primary samples. Created with BioRender. **b** Overview of RMS sample cohort, including patient clinical characteristics, as well as a summary of relevant mutations and copy number variants (CNV) in tumours, defined using bulk DNA sequencing. ( + ) and (#) indicate independent samples derived from the same patient. **c** Representative haematoxylin and eosin (H&E) stained tumour sections depicting the two major RMS histological subtypes (alveolar and embryonal) in this cohort. Scale bars are equivalent to 200 µm. Images representative of stained sections from all samples in the primary cohort (*n* = 19) **d** UMAP projection of single-cell RMS transcriptomes from primary samples (*n* = 7364) coloured by sample. **e** Dot plot depicting the average scaled gene expression of selected marker genes for each annotated cell type (dot colour). Dot size corresponds to the percentage of cells expressing each gene. **f** Boxplots comparing the proportion of malignant cells (left panel) and each non-malignant cell type (right panel) between molecular subtypes (*n* = 17 biologically independent samples). ns = not significant (*p* > 0.05, two-sided student’s *t* test). Both panels exclude bone marrow aspirate samples. The mean is used as the centre measurement for each box, which encloses the range between the first and third quartiles. Whiskers extend to the largest (or smallest) values no further than 1.5× the inter-quartile range (IQR) from the box hinges. Source data are provided as a Source Data file.

We implemented a protocol for performing plate-based single-cell mRNA-sequencing^15^ (SORT-seq) on viably frozen primary RMS tumour samples and recently established patient-derived tumour organoid models^16^ (Fig. 1a and Supplementary Fig. 1a,c). Opting for a plate-based method allowed for the generation of high-quality single-cell transcriptomes from primary samples with low viability (including pre-treated samples) or where limited material was available (e.g. small needle biopsies). From our cohort of 27 RMS samples (19 primary samples and 8 previously established tumour organoid models^16^), encompassing the major molecular and histological subtypes (FP, FN, alveolar and embryonal, Fig. 1b,c, Supplementary Fig. 1b and Supplementary Data 1), we obtained 10,216 high-quality single-cell transcriptomes which passed quality thresholds (median of 420 per primary sample and 319 per tumour organoid).

To distinguish RMS cells in primary samples from non-malignant cell types comprising the TME, two complementary approaches were employed. First, the similarity between each single-cell transcriptome and a reference collection of bulk transcriptomes derived from healthy cell types and RMS tumours was assessed using SingleR^17^ (see Methods). Clustering of the resulting similarity scores revealed a clear distinction between cells with a high correspondence to bulk RMS tumours (malignant cells) and those which resembled one of several immune or stromal cell types (Supplementary Fig. 1d). Second, single-cell copy number variant (CNV) profiles were inferred and clustered on a per tumour basis. In all tumours, cells harbouring coherent whole and sub-chromosomal CNVs (malignant cells) could be distinguished from those which appear to be copy neutral (Supplementary Fig. 2). In general, single-cell derived CNV profiles were highly similar to those defined by whole genome sequencing of bulk tumour samples^16^ (Supplementary Fig. 2). Cells classified as “malignant” or “normal” using both methods were retained, while divergently classified cells were excluded from further analysis ( < 2% of total primary cells, Supplementary Fig. 1e). The median percentage of malignant cells per primary sample was 56%, though this varied widely (2%-97%) and did not differ significantly between molecular subtypes (Fig. 1f and Supplementary Fig. 1e). As expected, putative malignant cells expressed high levels of classical RMS marker genes *DES, MYOD1* and *MYOG* (Fig. 1e). SingleR cell-type similarity scores and the expression of known marker genes were then used to discern the identities of non-malignant cells (Fig. 1e and Supplementary Fig. 1b). As with the overall percentage of malignant cells, the proportion of each non-malignant cell type varied extensively between tumours but did not differ significantly based on fusion status (Fig. 1f and Supplementary Fig. 1e). Projecting the classified primary single-cell transcriptomes in Uniform Manifold Approximation and Projection (UMAP) space revealed that inter-tumoral heterogeneity and molecular subtype classification (FN or FP) drove the clustering of malignant cells, while non-malignant cells clustered by cell type (Fig. 1d), as has previously been described for other tumour entities^18–21^. Clustering based on molecular subtype and inter-tumoral heterogeneity was also observed for the tumour organoid cells, supporting the presence of subtype-specific transcriptomic differences that are retained after in vitro expansion (Supplementary Fig. 1g).

### Characterisation of the RMS microenvironment reveals general and subtype-specific immune dysfunction

**Fig. 2:**
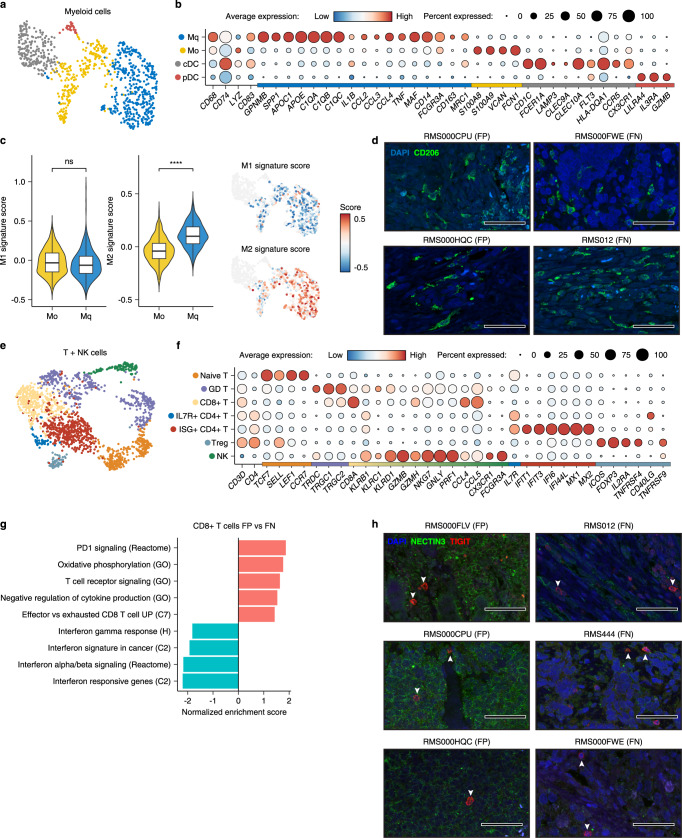
Characterisation of the RMS immune microenvironment. **a** UMAP projection of myeloid cells, coloured by cluster assignment. **b** Dot plot depicting the average expression of selected cell type-specific genes (Mq = differentiated macrophages, M0 = undifferentiated macrophages, cDC = conventional dendritic cells and pDC = plasmacytoid dendritic cells). Dot size corresponds to the percentage of cells expressing each marker. Colour bar on the *x*-axis indicates for which cluster each gene is specific. **c** Combined Violin/Box and UMAP plots showing the distribution of M1 (left panel) and M2 (right panel) signature scores in undifferentiated (M0, coloured yellow in the violin/box plots) and differentiated (Mq, coloured blue in the violin/box plots) macrophages (*n* = 637 biologically independent cells). ns = not significant (*p* > 0.05, Student’s *T* test), **** indicates *p* < 2.2e−16 (two-sided student’s *T* test). Mean is used as the centre measurement for each box, which encloses the range between the first and third quartiles. Whiskers extend to the largest (or smallest) values no further than 1.5× the IQR from the box hinges. Non-macrophage cells are coloured grey in UMAP plots. **d** Representative immunofluorescence (IF) microscopy images depicting the expression of CD206 (green) and DAPI counterstaining (blue), in RMS tissue sections from FN and FP tumours. Scale bars equivalent to 50 µm. **e** UMAP projection of T and NK cells, coloured by cluster assignment. **f** Dot plot depicting the average expression of selected cell type-specific genes (Naïve T = Naïve T cells, GD T = Gamma delta T cells, CD8 + T = Cytotoxic T cells, ILR7 + CD4 + T = IL7R + T helper cells, ISG + CD4 + T = Interferon stimulated T helper cells, Treg = T regulatory cells, NK = Natural Killer cells). Dot size corresponds to the percentage of cells expressing each marker. Colour bar on the *x*-axis indicates the cluster specificity for each gene. **g** Normalised enrichment scores (NES) of selected gene sets, as determined by gene set enrichment analysis (GSEA) comparing CD8 + T cells between RMS subtypes. Codes in parenthesis indicate the database from which the gene set derives (H, C2 and C7 correspond to MSigDB collections). Colour corresponds to a positive (pink) or negative (blue) NES. **h** Representative IF microscopy images depicting the expression of TIGIT (red) and NECTIN3 (green), along with DAPI counterstaining (blue), in RMS tissue sections from FN and FP tumours. White arrows highlight TIGIT+ cells. Scale bars equivalent to 50 µm. Source data are provided as a Source Data file.

To explore the composition and functional characteristics of tumour-infiltrating immune cells, graph-based clustering was performed on the myeloid and T/NK compartments of primary RMS samples (Fig. 2a,e). Examination of marker gene expression across myeloid clusters revealed the presence of undifferentiated (M0) and differentiated (Mq) macrophages, as well as conventional (cDC) and plasmacytoid (pDC) dendritic cells (Fig. 2b and Supplementary Fig. 3a). Scoring differentiated macrophages for M1/M2-specific gene signatures^22^ (listed in Supplementary Table 1) indicated that they existed predominantly in the M2 polarisation state (Fig. 2c), which is associated with several pro-tumorigenic functions including the suppression of inflammation and promotion of angiogenesis^23^. This finding was supported by immunofluorescence (IF) microscopy on primary tumour tissue showing infiltration of CD206+ and CD163+ cells across multiple patient samples (Fig. 2d and Supplementary Fig. 3b).

Among the T/NK cell clusters, several subtypes could be discerned including naïve and gamma delta (GD) T cells, regulatory T cells (Tregs), cytotoxic (CD8 + ) T cells and multiple subtypes of CD4 + T helper cells (IL7R+ and ISG + ) (Fig. 2f and Supplementary Fig. 3c). IHC for immune cell markers confirmed the presence of infiltrating T cells in RMS tissues (Supplementary Fig. 1f). Interestingly, interferon-stimulated T helper cells (ISG + ) were found almost exclusively in FN tumours, which may reflect a higher degree of immunogenicity (Supplementary Fig. 3b). Within T cell subgroups, the expression of several genes encoding molecules associated with immune dysfunction and the suppression of immune responses^24^ was observed, including *LAG3* and *PDCD1* (PD1) in CD8 + T cells, *CTLA4* and *TIGIT* in Tregs and *HAVCR2* in NK cells (Supplementary Fig. 3c). Strikingly, gene set enrichment analysis (GSEA) comparing CD8 + T cells between RMS subtypes indicated that dysfunction was more prevalent in FP samples, which were enriched for gene sets related to PD-1 signalling, oxidative phosphorylation and T cell exhaustion, while cells from FN tumours were enriched for interferon response and stimulation signatures (Fig. 2g). To define putative cell-cell interactions regulating immune dysfunction, we used CellChat^25^ to model ligand-receptor interactions between malignant cells, per subtype, and cell types within the TME. This analysis highlighted a putative interaction, specific to FP tumours, between NECTIN3 expressed on malignant cells and the TIGIT receptor on Tregs and CD8 + T cells (Supplementary Fig. 3e). The specificity of this interaction was due to the significantly higher expression of *NECTIN3* in FP tumour cells, while the expression of *TIGIT* in Tregs and CD8 + T cells was comparable between subtypes (Supplementary Fig. 3f). Supporting this finding, IF microscopy on primary tumour tissue revealed the presence of TIGIT-positive cells in both subtypes, while a consistently more prevalent staining pattern of NECTIN3 was observed in FP RMS (Fig. 2h). Taken together, analysis of the TME in RMS highlighted evidence of general immune dysfunction, as indicated by the prevalence of M2 polarised macrophages, as well as a putative FP-specific T-cell exhaustion phenotype which may in part be regulated by the interaction between NECTIN3 and TIGIT.

### Malignant cell states in RMS mirror normal myogenic differentiation

While it has been proposed that RMS tumours arise as a result of myogenic differentiation gone awry, the identification of the precise developmental origin(s) of RMS remains an active area of investigation^26^. To place primary RMS tumour cells within the context of normal myogenic differentiation, a series of logistic regression models were trained, as previously described^27^, to predict the similarity of malignant single-cell transcriptomes to the main cell types defined by a recently published single-cell atlas of human pre- and post-natal myogenesis^28^. This analysis showed that, on average, FN RMS cells resembled both myogenic progenitors and myogenic mesenchymal cells, while FP cells most closely corresponded to committed myoblasts (Supplementary Fig. 4a). This is in line with the notion that FN tumours often exhibit an undifferentiated “embryonal” histology, while FP more widely express the key myogenic regulatory factors responsible for orchestrating terminal differentiation, *MYOD1* and *MYOG*^6^ (Fig. 1b,c and Supplementary Fig. 4b). However, when analysing at single-cell resolution we found that individual cells from each subtype and tumour spanned the spectrum of myogenic differentiation, indicating that there exists large-scale intra-, as well as inter-tumoral heterogeneity in cellular differentiation states (Supplementary Fig. 4a).

### NMF-defined differentiation trajectories in FN RMS reflect early myogenesis

**Fig. 3:**
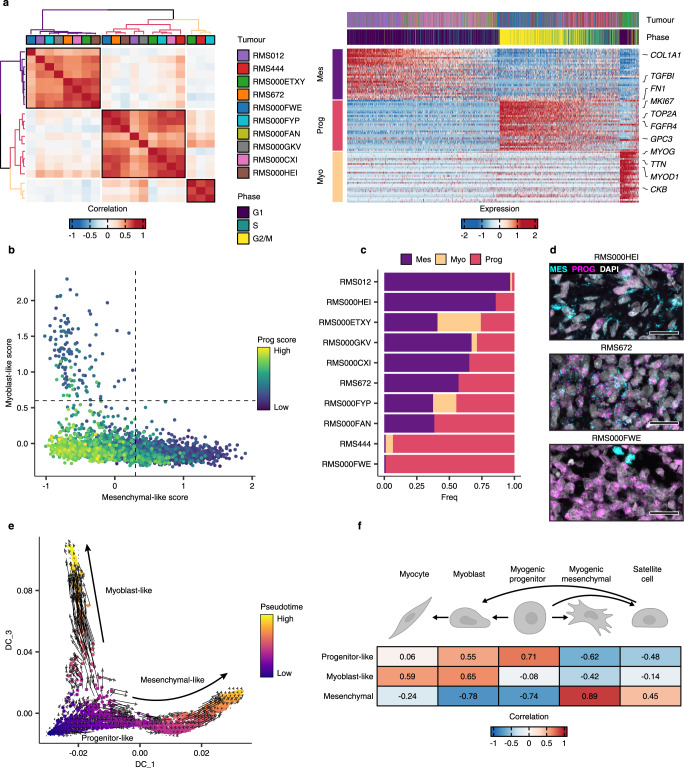
NMF defines malignant cell states in FN RMS tumours. **a** Left panel: Heatmap showing the pairwise Pearson correlations between all NMF-defined transcriptional programs in FN samples. The tumour sample from which each transcriptional program was derived is shown in the colour bar. Meta-program clusters are delineated by black boxes and colouring of the dendrograms. Right panel: Scaled expression of the top 30 genes per meta-program across all FN cells (Myo = Myoblast-like, Prog = Progenitor-like and Mes = Mesenchymal-like). The corresponding tumour sample and inferred cell cycle phase of each cell are displayed in the top annotation track. Representative genes from each meta-program are labelled. **b** Scatterplot depicting the mesenchymal-like (*x*-axis), myoblast-like (*y*-axis) and progenitor-like (point colour) meta-program scores. Dotted lines correspond to the cut-offs used to define discrete cell states. **c** Proportion of cells within each discrete state, per FN tumour. **d** Representative RNA fluorescence in-situ hybridisation (RNA-FISH) images depicting the expression of mesenchymal-like (MES = *TGFBI*) and progenitor-like (PROG = *FGFR4*) cell state marker genes in FN tissue samples. DAPI counterstaining is shown in grey. Scale bars equivalent to 25 µm. **e** Diffusion maps projection of FN RMS single cells, coloured by pseudotime value, overlaid with the RNA velocity vector field. **f** Heatmap depicting the Pearson correlations between cell-state scores, and the logistic regression-defined similarity scores (logits) for each normal myogenic cell type. Myogenic differentiation schematic was created with BioRender. Source data are provided as a Source Data file.

To probe the prospective sources of heterogeneity, non-negative matrix factorisation (NMF) was applied, independently per molecular subtype, to define the underlying transcriptional programs active in malignant cells from each of the primary tumours in our RMS scRNA-seq cohort (see Methods). In FN RMS samples, this analysis revealed three clusters of highly correlated transcriptional programs, which we merged into three meta-programs (Fig. 3a-left panel). Notably, the constituent programs underlying each meta-program were derived from several tumour samples, indicating that clustering was not driven by inter-tumoral differences. To interpret the biological relevance of each meta-program, we assessed the expression of their top-weighted genes (Fig. 3a-right panel, Supplementary Table 2 and Supplementary Data 2). The first program, which we termed “mesenchymal-like”, was enriched for genes related to extracellular matrix (ECM) organization, including *FN1*, *TGFBI* and several collagen-encoding genes, among others (Supplementary Fig. 5a). The second program, referred to as the “progenitor-like” program, included genes expressed during early myogenesis^28^, such as *FGFR4* and *GPC3*, as well as markers of proliferation, including *MKI67* and *TOP2A*. As this suggests, cells with high expression of progenitor-like program genes were inferred to have high cell cycle activity (Fig. 3a-right panel). Finally, the “myoblast-like” program was characterised by genes involved in the regulation of terminal myogenic differentiation, including *MYOD1*, *MYOG*, *MEF2C* and *CDH15* as well as genes encoding structural and functional components of terminally differentiated striated muscle, such as *TTN* and *CKM*. Scoring FN cells for each meta-program revealed that expression of the myoblast-like and mesenchymal-like programs was mutually exclusive, while expression of the highly proliferative progenitor-like state was restricted to cells that scored low for the mesenchymal-like as well as the myoblast-like programs (Fig. 3b). These associations were corroborated using a dataset from a recently published independent single-nucleus RNA-seq cohort of RMS tumours^29^ (Supplementary Fig. 5b).

Meta-program scores were then used to define the discrete “state” of each cell. This analysis revealed a high degree of variation between tumours in the distribution of cell states (Fig. 3c). Interestingly, some tumours (e.g. RMS012 and RMS000HEI) were dominated by mesenchymal-like cells, while others (e.g. RMS444 and RMS000FWE) almost exclusively contained progenitor-like cells. RNA fluorescence in-situ hybridisation (RNA-FISH) was used to validate the presence of each cell state and the distribution of the progenitor-like and mesenchymal-like states within individual tumours (Fig. 3d and Supplementary Fig. 5c). To investigate the hierarchy of cell states in FN RMS, the data were modelled as a differentiation trajectory by projecting single-cell transcriptomes in diffusion maps space and using pseudotime and RNA velocity to assess directionality (Fig. 3e and Methods). This analysis suggested that cells transition from the highly proliferative progenitor-like state into the more differentiated mesenchymal-like or myoblast-like states. Variation in differentiation status was also evident when comparing the malignant cell-state scores with the similarity scores to normal myogenic cell types. This showed that the progenitor-like score correlated strongly with undifferentiated myogenic progenitors, while the mesenchymal-like and myoblast-like scores with more differentiated normal cell types, namely myogenic mesenchymal cells, and myoblasts/myocytes, respectively (Fig. 3f). We then leveraged the SCENIC pipeline^30^ to investigate the relationship between transcription factor (TF) activity and trajectory-specific pseudotime (see Methods). This analysis showed that as cells progress towards a mesenchymal-like state, the activity of several TFs known to play a role in the epithelial-to-mesenchymal transition (EMT), including TWIST1 and ZEB1 increased in activity (Supplementary Fig. 5d,e). Conversely, cells progressing towards a myoblast-like state exhibited upregulation in the activity of key regulators of terminal myogenic differentiation, including MYOD1, MYOG and MYF6 (MRF4). Finally, the highly proliferative progenitor-like cells (early along pseudotime in both trajectories) displayed an increase in the activity of cell cycle progression regulators, including several E2F TFs, as well as the myogenic progenitor marker SOX8. Together, these data show that transcriptional cell states in FN RMS cells can be organised in a differentiation trajectory mirroring that of early myogenic differentiation, where progenitor-like cells can give rise to cells resembling terminally differentiating myoblasts, or those progressing towards a mesenchymal-like state reminiscent of myogenic mesenchymal cells.

### Differentiation states in FP RMS mirror skeletal muscle regeneration

**Fig. 4:**
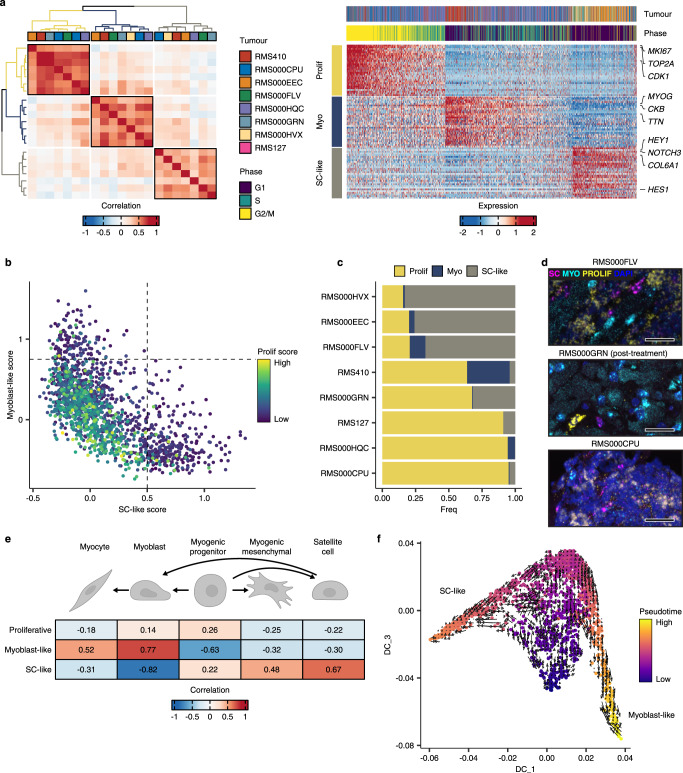
Cell states in FP RMS tumours mirror skeletal muscle myogenic differentiation. **a** Left panel: Heatmap showing the pairwise Pearson correlations between all NMF-defined transcriptional programs in FP samples. The tumour sample from which each transcriptional program was derived is shown in the colour bar. Meta-program clusters are delineated by black boxes and colouring of the dendrograms. Right panel: Scaled expression of the top 30 genes per meta-program across all FP cells (Myo = Myoblast-like, Prolif = Proliferative and SC-like = Satellite cell-like). The corresponding tumour sample and inferred cell cycle phase of each cell are displayed in the top annotation bar. Representative genes from each meta-program are labelled. **b** Scatterplot depicting per cell meta-program scores. Dotted lines correspond to the cut-offs used to define discrete cell states. **c** Proportion of cells within each discrete state, per FP tumour. **d** Representative RNA fluorescence in-situ hybridisation (RNA-FISH) images depicting the expression of satellite cell-like (magenta, SC = *NOTCH3*), myoblast-like (cyan, MYO = *TTN*) and proliferative (yellow, PROLIF = *MKI67*) cell state marker genes in FP tissue samples. DAPI counterstaining shown in blue. Scale bars equivalent to 25 µm. **e** Heatmap depicting the Pearson correlations between FP cell-state scores, and the logistic regression-defined similarity scores (logits) for each normal myogenic cell type. **f** Diffusion maps projection of FP RMS single cells, coloured by pseudotime value, overlaid with the RNA velocity vector field. Myogenic differentiation schematic was created with BioRender. Source data are provided as a Source Data file.

Extending the NMF analysis revealed three meta-programs specific to primary FP RMS, as defined by correlating transcriptional programs across tumour samples (Fig. 4a-left panel). The proliferative program consisted almost entirely of genes involved in mitotic cell processes, including *MKI67*, *TOP2A* and *CENPE*, among others (Supplementary Fig. 6a). As expected, nearly all cells inferred to be in S or G2/M phases scored high for this meta-program (Fig. 4a-right panel, Supplementary Table 3 and Supplementary Data 3). The myoblast-like program, like that found in FN RMS tumours, was marked by the expression of terminal myogenic differentiation genes, such as *MYOG*, *TTN* and *CKB*. Finally, the program termed “satellite cell-like” (SC-like) was characterised by the expression of the *NOTCH3* receptor gene, Notch pathway targets, including *HEY1* and *HES1*, and genes encoding type V and VI collagens. These genes are known to play roles in the context-specific regulation of quiescence, self-renewal, and activation in muscle-resident satellite cells^31,32^. Scoring single cells for each meta-program revealed a mutually exclusive relationship between the myoblast-like and SC-like programs, while the proliferative program did not correlate with either and was, in general, restricted to cells scoring low for the two former programs (Fig. 4b). Again, the relationship between meta-program scores was confirmed in an independent dataset^29^ (Supplementary Fig 6b).

As with the FN samples, there was a high degree of variation in discrete cell-state proportions between tumours, particularly among the proliferative and SC-like states (Fig. 4c). The expression of each meta-program, as well as the mutual exclusivity of the myoblast-like and SC-like programs, was validated using RNA-FISH (Fig. 4d). Comparisons between meta-program scores and the logistic regression-defined cell similarity scores showed that the myoblast-like program correlated strongly with cell types undergoing terminal differentiation (myoblasts and myocytes) while the SC-like program was most comparable to post-natal satellite-cells (Fig. 4e). The proliferative program score did not strongly correlate with any of the normal myogenic cell types, supporting the notion that this program was indicative only of cell cycle activity. Trajectory inference indicated that cells scoring high for the myoblast -like or SC-like programs lay at opposite ends of the differentiation continuum, while the proliferative cells appeared as an undifferentiated intermediate state (Fig. 4f). In this case, however, the RNA velocity results did not definitively imply a strict directionality of the trajectory (Fig. 4e). Finally, TF activity analysis showed that cells progressing towards a myoblast-like state had high activity of MYOG and MYOD1, while cells in the proliferative state were marked by high E2F TF activity (Supplementary Fig. 6c, d). Notably, in addition to high activity for the NOTCH pathway effector TF HEY1, the SC-like state was associated with an upregulation in the activity of the key satellite cell regulator PAX7, further supporting the resemblance between the SC-like cell state in FP RMS and normal satellite cells. Altogether, these data showed that the shared cell-state heterogeneity in FP RMS forms a differentiation trajectory reminiscent of that underlying skeletal muscle regeneration, where SC-like cells connect to cells resembling terminally differentiating myoblasts through a proliferative, undifferentiated cell state.

### Validation of subtype-specific malignant cell states in tumour organoid models of RMS

To determine whether the subtype-specific cell states defined above and validated in an independent cohort of RMS samples^29^ are tumour cell intrinsic, we sought to decouple the influence of the TME from malignant cells. It was previously demonstrated that RMS tumour organoid models, shown above to display the molecular-subtype-specific clustering observed in primary malignant cells (Supplementary Fig. 1g), faithfully recapitulate RMS tumours^16^. As RMS tumour organoids are composed solely of malignant cells, we reasoned that if the intra-tumoral heterogeneity observed in the primary tumours is tumour cell intrinsic, it would be reflected in these models. To investigate this, we scored tumour organoid cells for each of the subtype-specific meta-programs defined using NMF. Among FN tumour organoid cells, the relationship between the 3 programs resembled what was observed in primary RMS tumours: expression of the mesenchymal-like and myoblast-like programs was mutually exclusive, while cells scoring highest for the progenitor-like program scored, in general, low for the other two programs (Supplementary Fig. 7a,b). Similarly, there was a strong negative association between the myoblast-like and SC-like programs in FP tumour organoid cells, whereas high scores for the proliferative program were found more frequently in cells scoring low for the two former (Supplementary Fig. 7c,d). Next, to assess whether patient-specific cell state heterogeneity was retained, we directly compared the meta-program scores between cells from primary tumours and derived tumour organoids for the 4 patients with matched samples. Overall, subtype-specific meta-program scores overlapped between the primary and tumour organoid samples (Supplementary Fig. 7e–h). In RMS012, cells from the tumour organoid generally scored lower for the mesenchymal-like program than primary tumour cells, which may be the result of in-vitro selection against the less proliferative mesenchymal-like state cells. Together, these data show that the subtype-specific cell states present in primary tumours are also reflected in RMS tumour organoid models, suggesting that this heterogeneity is indeed largely intrinsic to malignant cells and not induced by the TME.

### Malignant cell states are predictive of patient outcomes

**Fig. 5:**
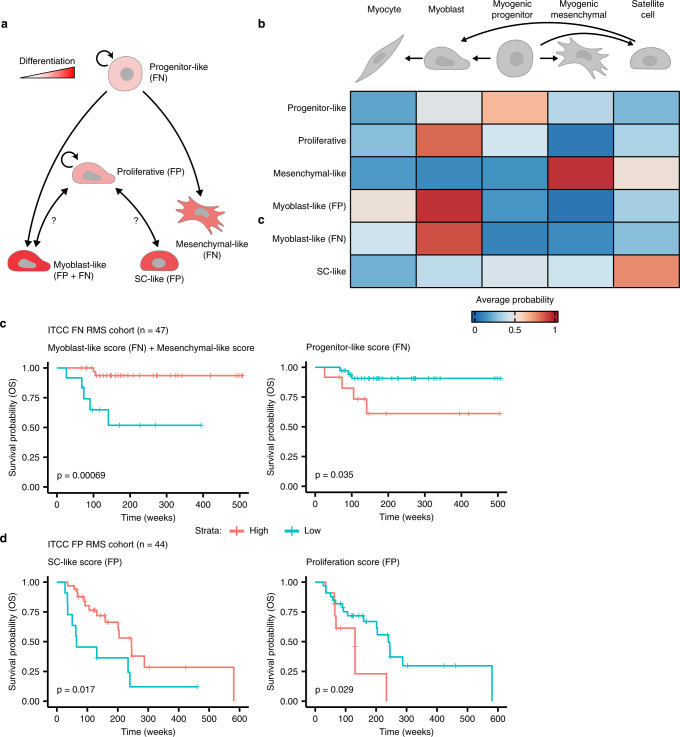
Malignant cell states are predictive of patient outcomes. **a** Schematic representation of differentiation trajectories in RMS. Created with BioRender. **b** Heatmap showing the average predicted similarity (probability) between discrete malignant cell states from both RMS molecular subtypes (y-axis) and normal myogenic cell types, as determined by logistic regression analysis. Myogenic differentiation schematic was created with BioRender. **c**, **d** Kaplan-Meier plots showing the overall survival probabilities of (**c**) FN (*n* = 47) or (**d**) FP (*n* = 44) patients divided into high (red strata) or low (blue strata) groups based on their cell state scores (stated in the title of each plot panels). Log-rank test was used to calculate *p* values between high- and low-scoring groups. Source data are provided as a Source Data file.

Taken together, results from the analysis of NMF-defined transcriptional programs allowed us to propose a unified model of cell states and differentiation trajectories in FN and FP RMS tumours (Fig. 5a,b). In FN tumours, highly proliferative cells with characteristics of early myogenic progenitors (progenitor-like) seem to give rise to cells which resemble either of two more differentiated types: myogenic mesenchymal cells (mesenchymal-like) or terminally differentiating myoblasts/myocytes (myoblast-like). In FP tumours, on the other hand, highly proliferative cells (proliferative) are an intermediate between cells closely resembling differentiating myocytes (myoblast-like), or post-natal skeletal muscle-resident satellite cells (SC-like). To investigate whether the differentiation state of RMS tumours affects their clinical behaviour, a published cohort of bulk tumour gene expression profiles^8^ was scored for each meta-program. Strikingly, FN RMS patients whose tumours had a high differentiation score (mesenchymal-like + myoblast-like) exhibited a significantly better OS probability than those with a low score (*p* = 0.00069, Fig. 5c-left panel). This result was particularly intriguing, as neither cell-state program was predictive of outcomes on its own (Supplementary Fig. 8a,b). Conversely, a high score for the undifferentiated progenitor-like program was indicative of significantly worse OS than FN tumours with a low score (*p* = 0.035, Fig. 5c-right panel). We compared the predictive power of the differentiation and progenitor-like scores with a previously validated meta-gene score for stratifying FN RMS patients (MG5.FN)^7^. While the distinction between high- and low-risk cases using the MG5.FN score was slightly more significant than with the progenitor-like score, the differentiation score predicted a group of patients with a significantly worse prognosis (Fig. 5c and Supplementary Fig. 8c). Notably, none of the genes present in the MG5.FN score overlapped with genes comprising any of the meta-program scores. In FP RMS patients, high expression of the SC-like program was associated with prolonged OS (*p* = 0.017), while a high proliferative score was indicative of shorter OS (*p* = 0.029, Fig. 5d). Differential expression of the myoblast-like program in FP tumours was not predictive of patient survival (Supplementary Fig. 8d). In summary, these data show that, in both RMS subtypes, tumours with higher proportions of cells in more differentiated states exhibit better outcomes than those with high levels of proliferative, less differentiated cells.

## Discussion

We generated a single-cell transcriptomic atlas of primary RMS tumours and patient-derived tumour organoid models, detailing cell states of both malignant cells and those constituting the TME. In our investigation of the TME, we found that, among differentiated macrophages in both RMS subtypes, the immunosuppressive, pro-tumour M2 polarisation state was predominant. Future work should focus on identifying the mechanism by which RMS tumour-associated macrophages are induced to an M2 polarisation, e.g. through cytokine secretion or signalling through direct cell-cell contact between RMS cells or other cell types in the TME. In addition, it will be important to understand the functional implications of tumour-associated M2 macrophages in RMS and determine whether polarisation can be impeded to encourage tumour-associated macrophages to take on a less immunosuppressive phenotype or exclude them from tumours entirely. Similar approaches are currently under clinical evaluation in other tumour entities^33^. We also described a putative interaction between FP tumour cells expressing NECTIN3 and the TIGIT receptor on Tregs and NK cells, which was supported by immunofluorescence staining of patient tissue samples. Interactions between TIGIT and several ligands, including NECTIN3, have been shown to suppress anti-tumour immune responses through several mechanisms^34^. As such, targeting this interaction may represent an opportunity to sensitise FP RMS tumours to immune-mediated killing using checkpoint inhibitors to block the TIGIT receptor^34^. Overall, we observed a higher proportion of T/NK cells, relative to myeloid cells (~1:1), than has been previously described in studies utilising immunohistochemistry^35,36^ or scRNA-seq^29^. Furthermore, beyond endothelial cells, we were unable to detect any other non-immune cell types in the TME, such as cancer-associated fibroblasts (CAFs) or non-malignant skeletal muscle cells. We ascribe these inconsistencies to biases introduced by the sample preparation protocol (e.g. the freeze/thaw cycle tumour samples were subjected to). However, including a larger number of T and NK cells had the benefit of allowing us to resolve and characterise functional subtypes not previously identified in RMS tumours.

In analysing NMF-defined transcriptional meta-programs and the similarity of RMS single-cells to normal myogenic differentiation, we defined subtype-specific hierarchies of malignant cell states. While directionality of the trajectories defined for FN RMS cells could be inferred, it was unclear in FP RMS whether SC-like cells derive from those in the proliferative state or “de-differentiate” and give rise to other cell states. It will be important, therefore, in future studies to examine the dynamic relationships between cell states in FP RMS using, for instance, phylogenetic analyses^37,38^ or functional assays in pre-clinical RMS models. Notably, our results illustrate the utility of RMS tumour organoid models in addressing questions such as these, as they share the presence of subtype-specific cell states observed in vivo. Our model of RMS differentiation trajectories has several clinical and biological implications. First, the observation that high levels of cells in more differentiated states are associated with better patient outcomes suggests the use of “differentiation therapy”, where tumour cells are pharmacologically induced to undergo differentiation^39^, would be a useful treatment strategy for RMS. In support of this, several studies using pre-clinical models of RMS have demonstrated that inhibiting critical pathways or regulators of tumorigenesis, including MEK in mutant RAS-driven FN RMS^40^ and BAF complexes in FP tumours^41^, leads to the induction of terminal myogenic differentiation. This approach could be expanded upon in future studies through the systematic elucidation of key regulators of RMS cell states which could be targeted to induce differentiation. On the other hand, the observation that high levels of proliferation are associated with worse outcomes supports the potential utility of compounds targeting key cell cycle regulators, including WEE1, PLK1 or CDK4/6 inhibitors, all of which are being investigated as therapeutic additions to RMS treatment regimens^42^. In addition to informing future treatment strategies, our results suggest that the differentiation state of RMS tumours could be a valuable metric for patient stratification, particularly in FN RMS. The translation of this finding could help advance a key goal of RMS clinical research: the de-intensification of treatment, where possible, to reduce toxicity and treatment-induced late effects^43^. However, these results first need to be validated in independent patient cohorts, an effort which is complicated by the overall lack of publicly available data sets combining gene expression of RMS tumours with clinical follow-up information.

Importantly, several of the cell states defined here using NMF are confirmed by findings detailed in other recent studies of RMS intra-tumoral heterogeneity. Patel et al.^29^ and Wei and Qin et al.^44^ also found the presence of highly proliferative cell states, as well as cells appearing to undergo terminal myogenic differentiation in both RMS subtypes. Likewise, both detected the presence of a largely quiescent population of cells, unique to FN RMS tumours, exhibiting mesenchymal characteristics (termed “mesoderm” cells in Patel et al.^29^), with Wei and Qin et al.^44^ also detailing their similarity to the recently identified SkM. Mesen cells (referred to here as myogenic mesenchymal cells). In Wei and Qin et al.^29^, these “mesenchymal-like” cells arose from proliferative or “ground” state cells in-vitro, consistent with the differentiation trajectory presented here, but could re-enter the cell cycle and give rise to other cell states under stress conditions. Their further finding that enriching for these cells improved the efficiency of tumour growth in mice, as well as data from Patel et al.^29^ showing that “mesoderm” cells were treatment resistant, was intriguing considering our finding that a high mesenchymal-like score (in combination with a high myoblast-like score) was predictive of better OS in FN RMS patients. Additional work will be required to decouple how the mesenchymal-like state influences outcomes in FN RMS.

In our comprehensive analysis of single-cell transcriptomes from paediatric RMS, we characterised the immune component of the TME and defined cell-states mirroring normal myogenic differentiation trajectories. Based on these findings, we propose that targeting immune checkpoint molecules and suppressive M2 macrophages are promising therapeutic approaches for RMS that merit further investigation. Furthermore, the validation and clinical implementation of differentiation state as a prognostic indicator should be a priority, given its potential to improve patient risk stratification.

## Methods

### Tumour sample acquisition

RMS tumour samples were obtained via an established sample acquisition route as part of the biobank initiative of the Princess Máxima Center for Pediatric Oncology, Utrecht, Netherlands (remaining tumour samples). Ethics approval was granted for the biobanking initiative by the Medical Research Ethics Committee (METC) of the University Medical Center Utrecht, and the Maxima biobank committee granted approval for the present project. All patients and/or their legal representatives signed informed consent to have tumour samples taken for biobank usage. Experiments conformed to the principles set out in the WMA Declaration of Helsinki and the Department of Health and Human Services Belmont Report.

### Tumour organoid samples

Tumour organoid models used in this study were established previously, as described in Meister et al.^16^.

### Sample processing and single-cell RNA-sequencing

Viably frozen primary tumour samples were rapidly thawed in a water bath, minced using a scalpel and then transferred to a tube containing 4.5 ml of BM1* medium (Advanced DMEM/F12 [Gibco, cat no. 12634010] supplemented with 1% Glutamax [Gibco, cat no. 35050061], 1% Penicillin/Streptomycin [Gibco, cat no. 15140122], 2% B27 minus vitamin A [Gibco, cat no. 12587010], 1% N2 [Gibco, cat no. 17502048], 0.25% N-acetylcysteine [500 mM, Sigma, cat no. A9165], 1% MEM non-essential amino acids [Gibco, cat no. 11140035], 1% sodium pyruvate [100 mM, Gibco, cat no. 11360070], 0.01% heparin [5000 U/ml, Sigma, cat no. H3149-10KU], 1% hEGF [2 µg/ml, Peprotech, cat no. AF-100-15], 0.1% hFGF-basic [40 µg/ml, Peprotech, cat no. 100-18B], 0.02% hIGF1 [100 µg/ml, Peprotech, cat no. 100-11], 0.01% Rho kinase inhibitor [Y-27632, 100 mM, AbMole Bioscience, cat no. M1817] and 0.1% A83-01 [5 mM, Tocris Bioscience, cat no. 2939]). To this, 0.5 ml of Collagenase D (Roche, #11088866001, 1:10 dilution) and DNAseI (Stemcell #07900, stock diluted 1:40 in PBS, further 1:100 diluted in the BM1* mixture) were added, and samples were allowed to dissociate in a shaker set to 250 rpm for 30 min at 37 °C. Following digestion, samples were passed through a 70 µm strainer which was subsequently flushed with an additional 5 ml of BM1* (supplemented with DNAseI) to increase the yield. Samples were then washed twice with 5 ml of washing medium (Advanced DMEM/F12 supplemented with 1% Glutamax, 1% Penicilin/Streptomycin and 1% HEPES [1 M, Gibco, cat no. 15630049]), centrifuging at 300 *g* for 5 min (at 4 °C) in between steps. After the final washing step samples were resuspended in BM1* (supplemented with DNAseI) to a final concentration of <1 × 10^6^ cells per ml. Viably frozen tumour organoid samples, generated previously in our lab^16^, were rapidly thawed in a water bath and immediately resuspended in BM1* (supplemented with DNAseI) to a final concentration of <1 × 10^6^ cells per ml after a washing step to remove the DMSO necessary for freezing. Prior to sorting, 4′,6-diamidino-2-phenylindole (DAPI, Sigma-Aldrich, #D9542) and DRAQ5 (Thermo Fisher, #65-0880-92) were added to single-cell suspensions up to final concentrations of 1 µM and 5 µM, respectively. Viable single-cells (DAPI−, DRAQ + ) were then sorted into 384-well plates containing 10 µl of mineral oil (Sigma, #M5310) and 50 nl of barcoded RT primers using a SONY SH800S Cell Sorter (SONY SH800S system software v2.1). Libraries were prepared according to the SORT-seq^15^ protocol and sequenced on an Illumina NextSeq500 (paired-end, 75 bp read chemistry) by Single Cell Discoveries B.V.

### Immunohistochemistry and H&E staining

Immunohistochemistry (IHC) and haematoxylin and eosin (H&E) staining experiments were performed on 4 µm thick formalin fixed and paraffin embedded (FFPE) tissue sections using a Ventana automated tissue staining system (BenchMark Ultra, Roche). For IHC, the antibodies used were anti-CD3 clone LN10 (Leica, PA0533), anti-CD8 4B11 (Leica, PA0183) and anti-CD68 514H12 (Leica, PA0273).

### Immunofluorescence microscopy

Mounted tumour sections (5 µm thick FFPE) were baked at 60 °C for 1 h, then deparaffinized and rehydrated using sequential washes of Xylene (2 × 100%), Ethanol (2 × 100%, 2 × 95%, 1 × 75%, 1 × 50% and 1 × 25%) and demineralised H_2_O (2 × 1 min, 1 × 5 min). Antigen retrieval was then performed by boiling slides in either Tris-EDTA, pH 9 (anti-TIGIT and anti-NECTIN3) or Sodium citrate, pH 6 (anti-CD206 and anti-CD163) for 20 min in a benchtop autoclave. Slides were then washed 3 × 5 min in PBST (PBS + 0.1% Tween 20) and incubated with blocking solution (PBST + 1% BSA) for 1 h at room temperature. After blocking slides were incubated with primary antibody diluted in blocking solution overnight at 4 °C. The following day, slides were washed 3 × 5 min with PBS and then incubated with secondary antibody, diluted in PBST, in the dark for 1 h at room temperature. Slides were washed an additional 3 × 5 min with PBS before adding mounting medium containing DAPI counterstain (Vector labs, H-1200) and applying glass coverslips. Images were acquired on a Leica SP8 confocal microscope (40×/1.3NA oil immersion objective), and maximum projections of Z-stacks were obtained using the FIJI software (v2.0.0-rc-69/1.52i)^45^. Primary antibodies used: anti-TIGIT (Cell Signaling, #99567, 1:500 dilution), anti-NECTIN3 (R&D systems, AF3064, 1:200 dilution of a 0.2 µg/µl solution in PBS), anti-CD206 (Cell Signaling, #91992, 1:200) and anti-CD163 (Abcam, ab182422, 1:200 dilution). Secondary antibodies used: Donkey anti-Goat Alexa 647 (Abcam, ab150131, diluted 1:1000) and Donkey anti-Rabbit Alexa 568 (Abcam, ab175470, diluted 1:1000).

### RNA fluorescence in-situ hybridisation (RNAscope)

RNA-FISH experiments were performed on 5 µm FFPE tissue sections using the RNAscope^TM^ Multiplex Fluorescent v2 kit (ACD bio), according to the manufacturer’s instructions. The following probes were used for hybridisation: Hs-MKI67-C3 (591771-C3), Hs-TTN (550361), Hs-NOTCH3-C2 (558991-C2), Hs-FGFR4-no-XMm-C2 (443431-C2) and Hs-TGFBI (478491). In addition, the following fluorescent dyes were used for detection (diluted 1:1500): Opal 520 (FP1487001KT), Opal 570 (FP1488001KT) and Opal 690 (FP1497001KT). Images were acquired on a Leica SP8 confocal microscope (40x/1.3NA oil immersion objective), and maximum projections of Z-stacks were obtained using the FIJI software (v2.0.0-rc-69/1.52i).

### Data processing and quality control

Sequencing reads were demultiplexed, mapped to the GRCh38v2020-A genome, available from 10× genomics (https://support.10xgenomics.com/single-cell-gene-expression/software/release-notes/build), and transcript counts were generated using the zUMIs pipeline (v5.6)^46^. Using the Seurat R package (v4.1.0)^47^, count tables (per plate) were then loaded in R (v4.1.0), merged and metadata fields were compiled. Single cells were excluded if they expressed <500 unique genes, <800 or >50,000 unique transcripts, contained a percentage of mitochondrial transcripts >50%, contained >1% haemoglobin gene transcripts or contained a ratio of intergenic to genic transcripts >2. The data were then log normalised using a scale factor of 10,000 transcripts, and the normalised data were scaled and centered. The top 2000 most variably expressed genes were defined using the *FindVariableFeatures* function in Seurat (default parameters), and their expression was used as input for principal component analysis (PCA). Finally, the first 50 principal components were used to project single-cell transcriptomes in 2-dimensional space using uniform manifold approximation and projection (UMAP, default parameters). The top variable features and scaled gene expression values were recalculated to run PCA for the tumour organoid samples, and the top 30 principal components were used to project in UMAP space. The cell cycle phase of each cell was inferred using the *CellCycleScoring* function implemented in Seurat, using the built-in gene lists.

### Module scoring

Module scores were calculated as implemented in the Seurat function *AddModuleScore*, taking into account 25 expression bins and 100 control genes per query gene.

### Cell type classification

To discriminate between malignant and healthy cells, we first used the SingleR R package (v1.6.1)^17^ to annotate single cells based on their similarity to reference bulk transcriptomes of healthy cells (Human Primary Cell Atlas data^48^) and RMS tumours (EGAD00001008467). We then used the InferCNV R package (v1.8.0)^49^ to define and cluster single-cell copy number variant profiles per tumour sample (default parameters, using an average expression threshold of 0.3 and standard deviation filter of 2). A SORT-seq dataset of cord blood mononuclear cells (CBMC’s) and other normal cell types was used as reference. CNV profiles derived from bulk DNA sequencing were plotted for comparison (see Supplementary Fig. 2), and single-cell clusters containing CNVs were manually selected and annotated as “malignant”. In the final annotation, cells were called malignant when they were classified as such using both approaches and cells which were divergently classified (labelled ambiguous) were excluded from further analyses. The broad cell-type of non-malignant cells was inferred from the hierarchical clustering of the similarity scores.

### Analysis of the immune microenvironment

To reach sharper biological distinctions between immune cell subsets, SCTransform^50^ normalisation was performed on the full dataset to normalise and scale the data for unbiased clustering. To further improve detailed immune cell sub-clustering, sample-specific gene expression was removed to reduce technical effects and enhance biological variation. Sample-specific genes were identified by differential gene expression analyses among tumour cells and immune cells separately and comparing the individual samples. Genes that were differentially expressed in both the tumour cells and immune cells of a specific samples were considered sample-specific noise and were removed from the variable gene list. To avoid clustering of cells based on specific cell processes, genes associated with sex (*XIST*, *TSIX*, and Y chromosome-specific genes), cell cycle phase, dissociation stress (heat shock proteins; GO:0006986), and activity (ribosomal protein genes; GO:0022626), were also removed from the variable gene list.

Healthy clusters were subset and clustered using 40 principal components and a resolution of 0.3 (Louvain algorithm) was used to define clusters of the main cell types. For in-depth analysis of the T and NK cells, the respective clusters were subset, and UMAP was re-run using 40 PCs and a resolution of 0.5 was used to define subclusters. For in-depth analysis of the myeloid compartment, SCTransfrom normalisation was re-run, sample-specific and cell process-specific genes were removed from the variable gene list and 6 PCs and a resolution of 0.3 was used to define subclusters.

### Immune cell type identification

Cluster annotation was performed using SingleR, using the Human Primary Cell Atlas reference dataset to annotate main cell types, and additionally using the Novershtern Hematopoietic Data^51^ and Monaco Immune Data^52^ reference datasets to annotate the immune cell (sub)clusters. Cell annotations were further refined by consulting cluster-specific (up-regulated) differentially expressed marker genes using Seurat’s *FindAllMarkers* function. The output genes were compared to known cell-type specific marker genes from previous studies^53–56^.

### Gene set enrichment analysis (GSEA)

For GSEA, differential expression analysis between two groups was performed using the *FindMarkers* Seurat function, using the following adjusted parameters: logfc.threshold = 0, min.pct = 0, min.cells.feature = 0, min.cells.group = 0. Genes were pre‐ranked by their Fold Change and GSEA was performed using the R package fgsea (version 1.20.0). Gene sets with an FDR < 0.25 were considered significantly enriched. Gene sets were obtained for MSigDB version 7.2 using the msigdbr R package (version 7.4.1).

### Ligand-receptor interaction analysis

The CellChat algorithm was applied as implemented in the CellChat R package (v1.0.0) to perform an unbiased ligand‐receptor interaction analysis, using the curated ligand‐receptor database of CellPhoneDB (RRID: SCR_017054)^57^.

### Logistic regression analysis

Determination of the similarity between RMS single-cells and normal myogenic cell types (given as a probability value) was estimated as previously described in ref. ^27^. Briefly, we obtained the data described in ref. ^28^ from the gene expression omnibus (GSE147457) and trained logistic regression models using the main myogenic cell type labels. Correlations between meta-program scores and normal myogenic cell types used the logit-transformed probability values.

### Non-negative matrix factorisation

Non-negative matrix factorisation (NMF) was carried out using the NMF R package (v0.23)^58^. For each RMS subtype (FN or FP), a list of shared variable features (*n* = 2000) was compiled using the *SelectIntegrationFeatures* function in Seurat. The expression of these genes was then scaled, per tumour, and used as input to determine the appropriate NMF rank, by running 50 iterations (Brunet algorithm) for ranks between 2 and 10 (default settings). The optimal rank was determined, per tumour, by manually assessing in cophenetic coefficients, dispersion values and silhouette scores between rank values. We then re-performed NMF at 250 iterations using the optimal rank value. Per subtype, pairwise Pearson correlation coefficients were calculated between NMF-defined transcriptional programs (across all tumours) and hierarchical clustering was used to determine groups. Highly correlated groups of programs were merged into meta-programs by averaging gene weights. Cell-state scores were by using the top 30 weighted genes per meta-program to calculate module scores. Discrete cell-states were determined through manual inspection of the distribution of cell-state module scores.

### Gene list enrichment analysis

Functional enrichment of gene lists was performed using the enrichR R package (v3.0)^59^ (default settings) using the Reactome 2016 database.

### Comparison with data from Patel et al.

Single-nucleus RNA-seq data from the manuscript of Patel et al.^29^, was downloaded from the Single-Cell Pediatric Cancer Atlas Portal (https://scpca.alexslemonade.org/projects/SCPCP000005) and loaded into Seurat. We inferred malignant cells using SingleR, as described for the data presented in this study and applied an additional cut-off of >800 unique transcripts for a cell to be considered valid. Data were then split by molecular subtype and module scores were calculated, as described above, using the top 30 genes per meta-program.

### Differentiation trajectory modelling

Modelling of differentiation trajectories was done, per subtype, by projecting cells in DiffusionMaps space using expression of the top 30 meta-program-specific genes (destiny R package v3.1.1)^60^. The top 3 diffusion components were then used as input for trajectory modelling and cell lineage inference using Slingshot (v2.0.0)^61^. RNA velocity analysis was performed using the scVelo python package (v0.2.2, python v3.7)^62^. Briefly, input data per subtype, was filtered to include only genes with 20 shared (spliced and un-spliced) counts and log normalised. First and second order moments were calculated per cell using expression of the top 30 meta-program-specific genes and 30 nearest neighbours. RNA velocity was then estimated using the stochastic model and vectors were overlaid on the DiffusionMaps projections.

### Transcription factor activity analysis

The estimation of transcription factor activity was performed as previously described using the pySCENIC implementation (v0.12.1) of the SCENIC pipeline^30^. For each RMS subtype, 10 SCENIC runs were performed using the unnormalized count data as input, along with the auxiliary input databases “motifs-v9-nr.hgnc-m0.001-o0.0”, “hs_hgnc_tfs” and “hg38__refseq-r80__10kb_up_and_down_tss.mc9nr” (accessed from https://resources.aertslab.org/cistarget/). We considered transcription factors only if they were identified in ≥8 independent runs and calculated activity per cell as the mean AUCell values across runs. We then used the tradeSeq R package (v1.10.0)^63^ to fit a negative binomial generalised additive model (NB-GAM) for each transcription factor (setting the number of knots to 5).

### Survival analysis

Microarray gene expression profiles and the accompanying clinical follow-up information for the ITCC RMS cohort^8^ was downloaded from the R2 genomics platform (R2: Genomics Analysis and Visualisation Platform (http://r2.amc.nl). Samples which did not exhibit either of the two main RMS histological classifications (alveolar or embryonal) were excluded. The data were divided based on fusion transcript status and *Z*-scores were calculated per gene. To generate meta-program scores, the average *Z*-score of the top 30 genes per meta-program (in the appropriate dataset) was calculated per tumour. Based on the distribution of scores, the “high” scoring groups (and vice versa) were defined as either the top 25% or 75% of tumours. The MG5.FN score was applied as described in Missiaglia et al.^7^ Briefly, the per patient (FN) score was calculated as the sum of the weighted expression (signed *Z*-score) of the 5 genes. The cohort was then divided into tertiles, with the top tertile corresponding to high MG5.FN expression and the bottom two with low expression. Survival models were generated using the survival R package (v3.2-11) and *p* values were calculated using a Log-Rank test.

### Statistics and reproducibility

Immunohistochemical, immunofluorescence and RNA-FISH stainings were carried out once per patient due to limited material availability. When interpreting imaging results, at least 3 randomly chosen fields were surveyed.

### Reporting summary

Further information on research design is available in the Nature Portfolio Reporting Summary linked to this article.

## Supplementary information

Supplementary Information

Description of Additional Supplementary Files

Supplementary Data 1

Supplementary Data 2

Supplementary Data 3

Reporting Summary

## Data Availability

The raw sequencing data generated in this study have been deposited in the European genome-phenome archive. The accession number for the single-cell RNA-sequencing data is EGAD00001009385 (“Single-cell mRNA-sequencing to generate a transcriptomic atlas of RMS”). To protect patient privacy, as required by law, access to the sequencing data deposited in the EGA is controlled by the Data Access Committee (DAC) of the Princess Maxima Center. All researchers can obtain access by submitting a project proposal to the DAC (biobank-2 [at] prinsesmaximacentrum [dot] nl). Requests will be handled within ~2 weeks. The DAC will also determine the length of permitted access. The publicly available whole genome sequencing data generated by Meister et al.^16^ and used in Supplementary Fig. 2 can be accessed with the identifier EGAD00001008466 (“WGS soft tissue sarcoma tumoroid biobank”)^16^. The publicly available bulk RNA-sequencing data generated by Meister et al.^16^ and used in Supplementary Fig. 1d can be accessed with the identifier EGAD00001008467 (“RNA-Seq soft tissue sarcoma tumoroid biobank”)^16^. The publicly available single-nucleus RNA-sequencing data of RMS tumours generated by Patel et al.^29^ and used in this study are deposited in the single-cell paediatric cancer atlas portal under accession number SCPCP000005^29^ The publicly available single-cell RNA-sequencing data of normal myogenic differentiation used in this study are deposited in the GEO repository under accession number GSE147457^28^ and were accessed from https://skeletal-muscle.cells.ucsc.edu. The publicly available ITCC RMS microarray dataset is deposited in the gene expression omnibus (GEO) under accession number GSE92689 and were accessed from the R2 Genomics Analysis and Visualisation Platform (http://r2.amc.nl). The GRCh38v2020-A reference genome used to map the single-cell RNA-seq data was downloaded from the 10× genomics website (https://support.10xgenomics.com/single-cell-gene-expression/software/release-notes/build). The publicly available Human Primary Cell Atlas data, Novershtern Hematopoietic data and Monaco Immune data were accessed using the celldex R package (v1.2.0, https://github.com/LTLA/celldex). The publicly available CellPhoneDB ligand-receptor interaction data was accessed from https://www.cellphonedb.org (RRID: SCR_017054). The publicly available auxiliary input databases for the SCENIC analysis were accessed from https://resources.aertslab.org/cistarget/. The processed data used in and generated by this study, including compiled count tables and processed R objects, have been made publicly available via Zenodo (10.5281/zenodo.7928694)^64^. The remaining data are available within the Article, Supplementary Information or Source Data file. Source data are provided with this paper.

## Source data

Source Data

## Code availability

All data analysis scripts have been made publicly available via Zenodo (10.5281/zenodo.7928694)^64^.
