# Supplementary material for: Single-cell transcriptomics reveals immune suppression and cell states predictive of patient outcomes in rhabdomyosarcoma
Source: Nat Commun. 2023 May 27;14:3074. doi: 10.1038/s41467-023-38886-8 (PMC10224926; doi:10.1038/s41467-023-38886-8)
Supplement: Supplementary file 1 — Supplementary Information [file 41467_2023_38886_MOESM1_ESM.pdf]

Supplementary Figure 1

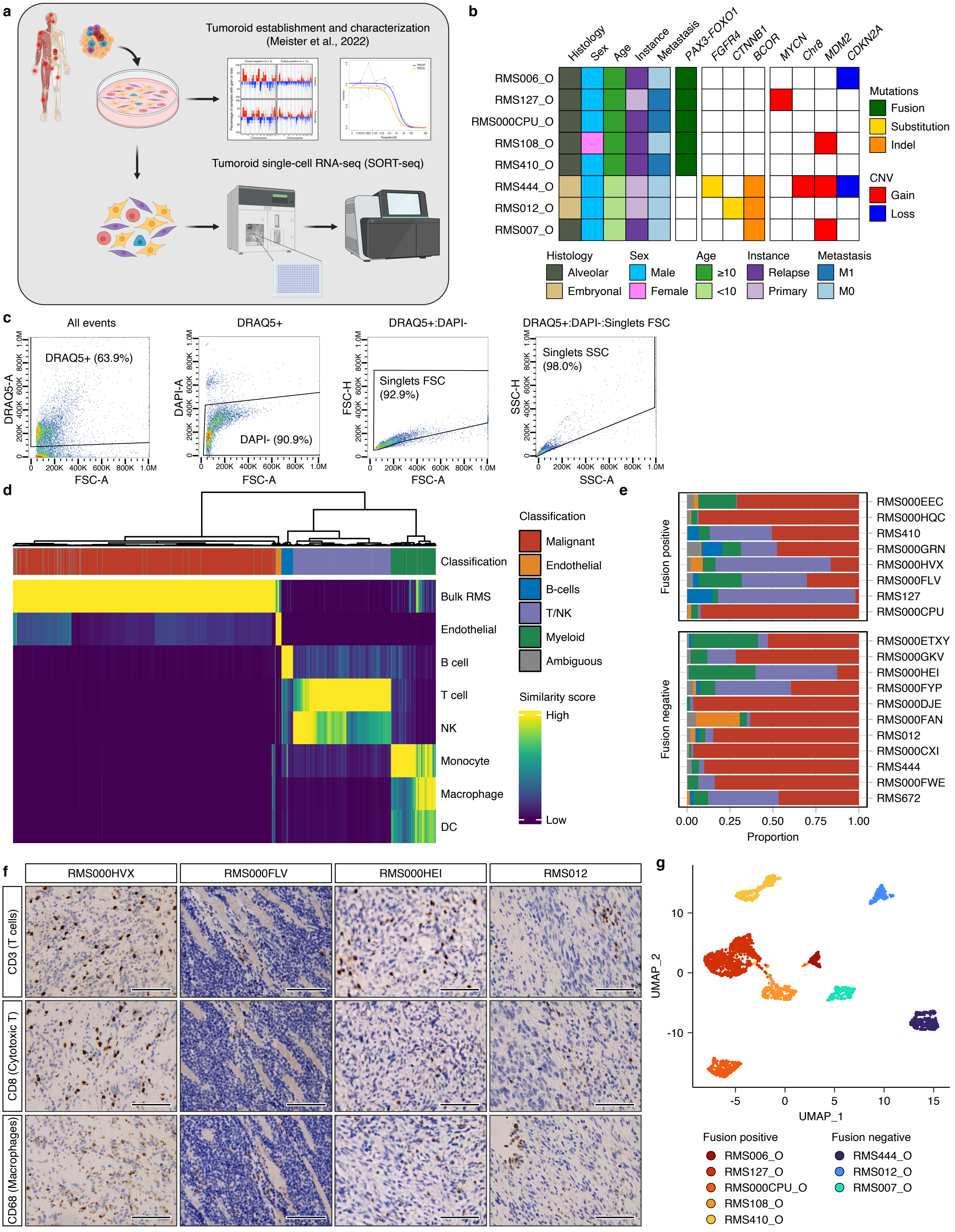

Supplementary Figure 1: Single-cell RNA-sequencing atlas of RMS tumours and tumour organoid models

a, Schematic representation of the sample processing workflow used to generate scRNA-seq data from tumour organoid samples. Created with BioRender. **b**, Overview of RMS tumour organoid sample cohort, including patient clinical characteristics, as well as a summary of relevant mutations and copy number variants (CNV) in tumours. **c**, Representative scatter plots showing the gating strategy used to sort single-cells for SORT-seq. Titles indicate the events shown in each plot (FSC = forward scatter, SSC = side scatter, -A = signal area and -H = signal height). **d**, Heatmap showing the clustered similarity scores (per primary cell) to each reference cell type (y-axis labels) as determined by SingleR. Cell classifications are shown in the top annotation bar. **e**, Bar graph depicting the distribution of annotated cell types per primary tumour sample. **f**, Representative microscopy images showing IHC staining of three immune population markers (y-axis) across primary tissue samples from four tumours (x-axis). Positive staining indicated by brown colouring, haematoxylin counterstained nuclei in blue. Scale bars equivalent to 100μm. **g**, UMAP projection of single-cell RMS transcriptomes from tumour organoid samples (n = 2,852) coloured by sample. Source data are provided as a Source Data file.

Supplementary Figure 2

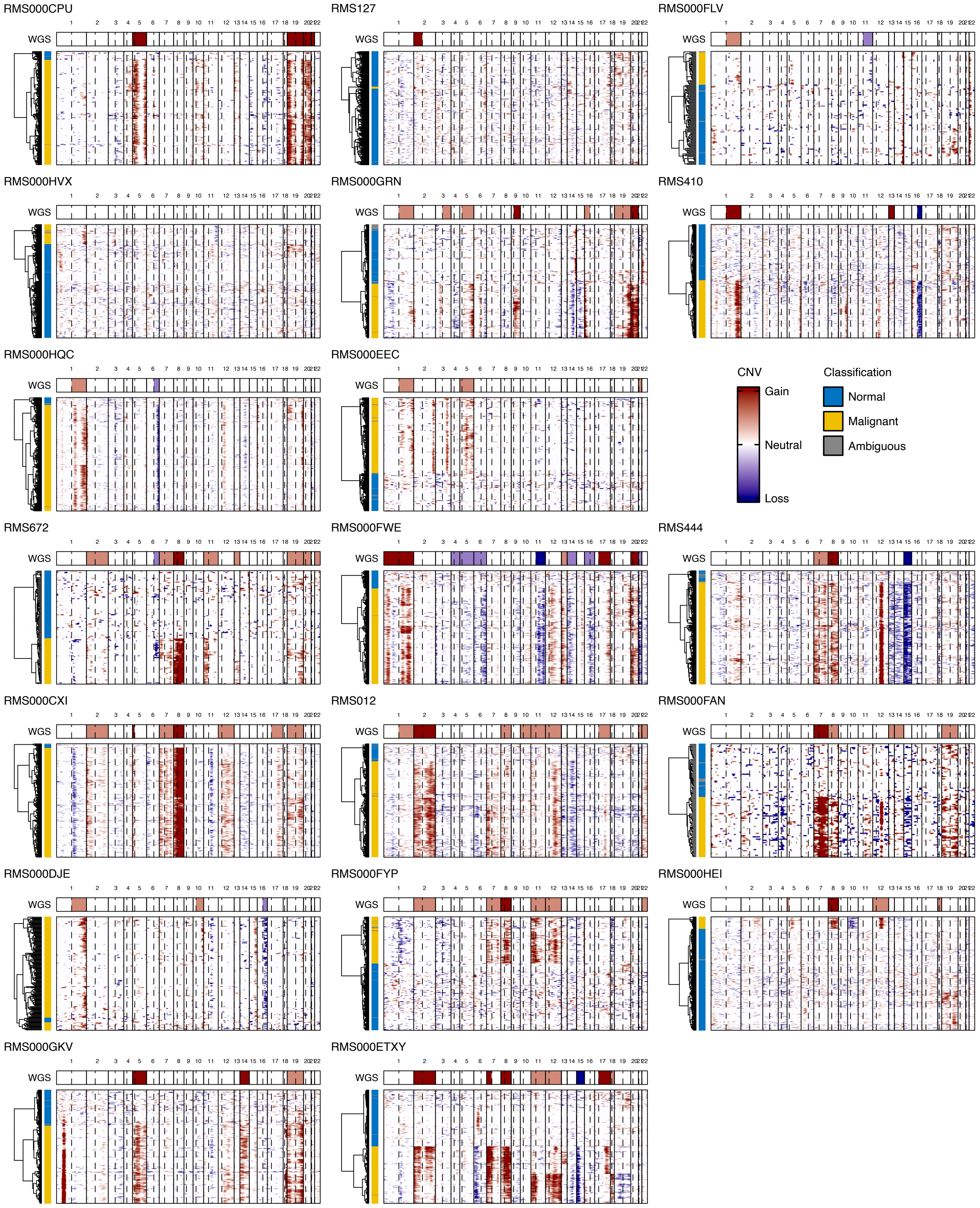

Supplementary Figure 2: Inferred CNV profiles of single-cell transcriptomes

Heatmaps showing the clustered single-cell inferred CNV profiles per tumour. Solid vertical lines denote chromosome boundaries and dotted vertical lines represent the locations of centromeres. Cell classifications are shown on the left annotation bars. Top annotation bars show CNVs (summarized per chromosome arm) defined by whole genome sequencing (WGS) of bulk tumour samples (from Meister et al.[1]), for comparison.

Supplementary Figure 3

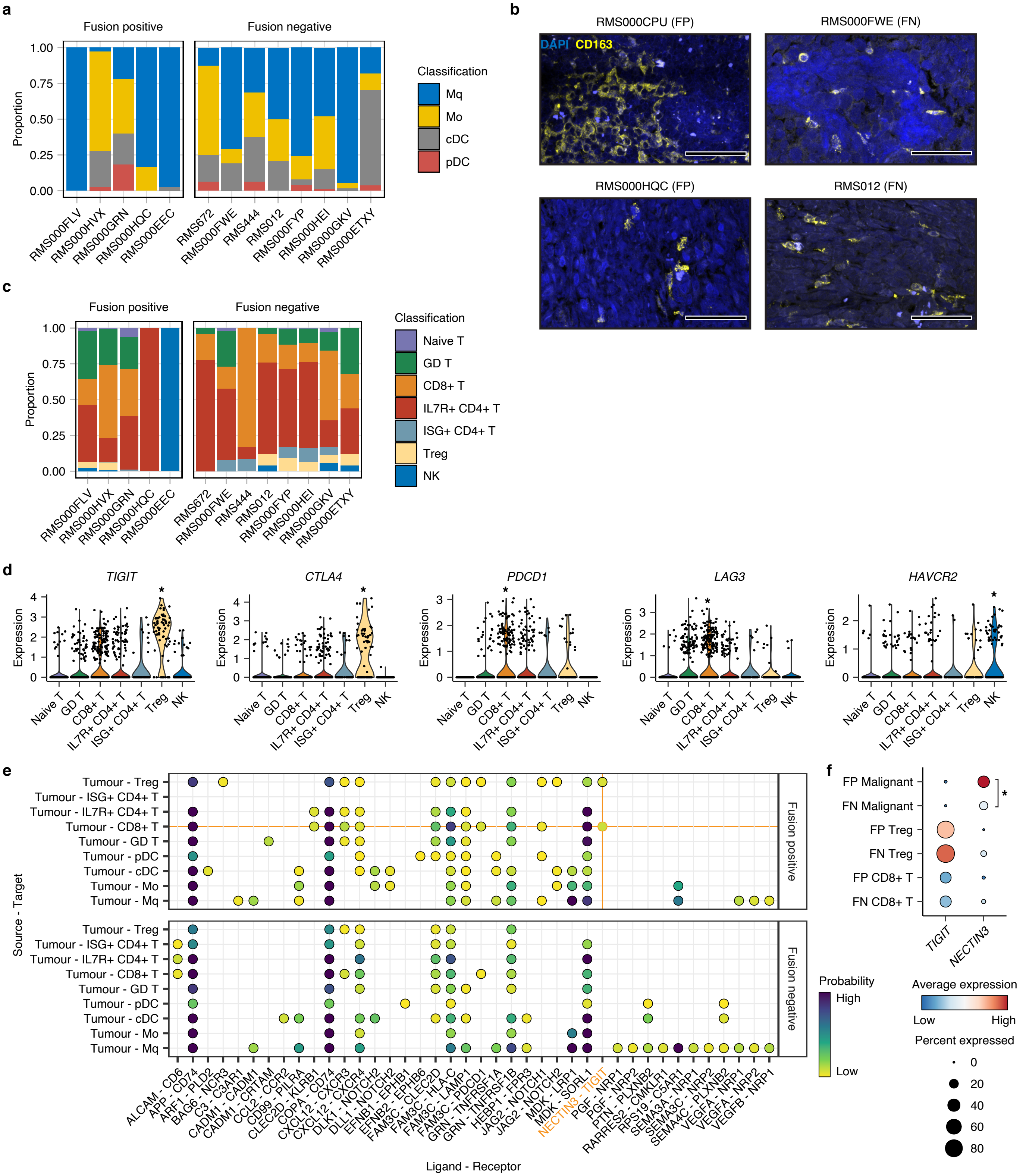

Supplementary Figure 3: Survey of the RMS tumour immune microenvironment

Bar plots showing the proportion of cell types within the (**a**) myeloid (Mq = differentiated macrophages, M0 = undifferentiated macrophages, cDC = conventional dendritic cells and pDC = plasmacytoid dendritic cells) or (**c**) T/NK (Naïve T = Naïve T cells, GD T = Gamma delta T cells, CD8+ T = Cytotoxic T cells, ILR7+ CD4+ T = IL7R+ T helper cells, ISG+ CD4+ T = Interferon stimulated T helper cells, Treg = T regulatory cells, NK = Natural Killer cells) compartments per tumour. Samples derived from bone marrow aspirates, or which contain less than 10 myeloid or T/NK cells are excluded. **b**, Representative immunofluorescence (IF) microscopy images depicting the expression of CD163 (yellow) and DAPI counterstaining (blue), in RMS tissue sections from FN and FP tumours. Scale bars equivalent to 50μm. **d**, Violin plots showing the expression of selected immune checkpoint molecules within T/NK subsets (n = 1720 biologically independent cells). Violin colour corresponds to cell type. * Indicates differential expression (Log2 FC > 0.25 and $p < 0.05$, two-sided Wilcoxon rank sum test with Bonferroni correction. Exact p values: *TIGIT* = 7.53e-52, *CTLA4* = 1.01e-49, *PDCD1* = 3.2e-22, *LAG3* = 1.97e-27 and *HAVCR2* = 5.32e-23). **e**, Dot plot summarizing the results of ligand-receptor interaction analysis, split by molecular subtype. Dots indicate an inferred ligand-receptor interaction (x-axis) between a source-target cell type pair (y-axis). Dots are coloured by the interaction probability, as determined by CellChat. The NECTIN3-TIGIT interaction is highlighted in orange on the x and y axes. **f,** Dot plot depicting the average expression of the *TIGIT* and *NECTIN3* genes in selected cell types (per RMS subtype). Dot size corresponds to the percentage of cells expressing each marker. * Indicates differential expression (LogFC > 0.25 and p = 2.52e-40, two-sided Wilcoxon rank sum test with Bonferroni correction, n = 4452 biologically independent cells). Source data are provided as a Source Data file.

Supplementary Figure 4

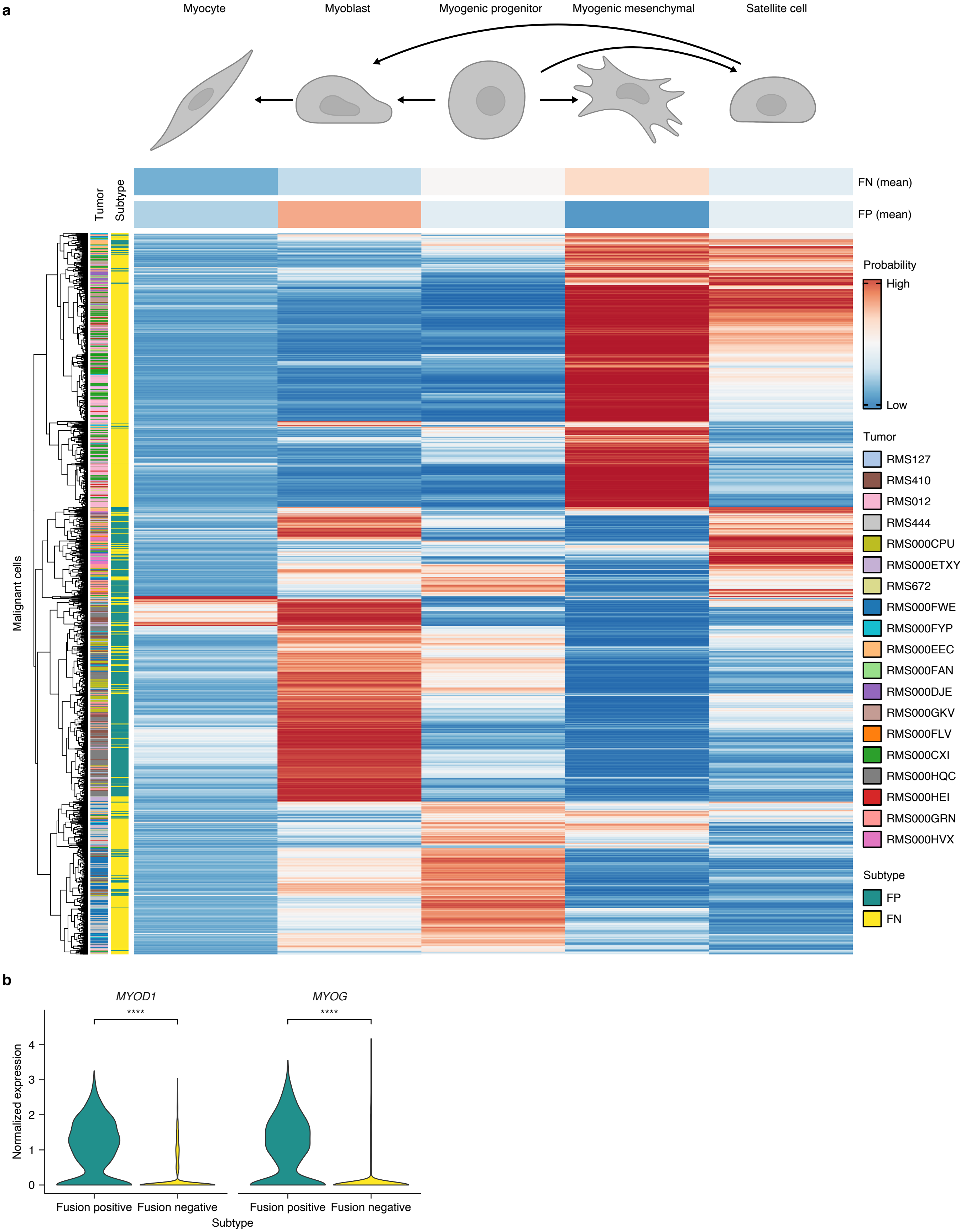

Supplementary Figure 4: Comparison between RMS tumours and normal myogenesis by logistic regression analysis

a, Heatmap showing the comparison between primary RMS single cells (rows) and normal myogenic cell types (columns). Colour values represent the predicted similarity (probability) as determined by logistic regression analysis. Annotation tracks (left) indicate the tumour and molecular subtype of each cell. The top two columns show the predicted similarity aggregated per molecular subtype. Myogenic differentiation schematic was created with BioRender. **b**, Violin plots depicting the normalized expression of *MYOD1* (left panel) and *MYOG* (right panel) in malignant cells from each RMS subtype (n = 8904 biologically independent cells). Colour corresponds to RMS molecular subtype. **** indicates differential expression ($p < 2.2e\text{-}16$, two-sided Wilcoxon Rank Sum test with Bonferroni correction). Source data are provided as a Source Data file.

Supplementary Figure 5

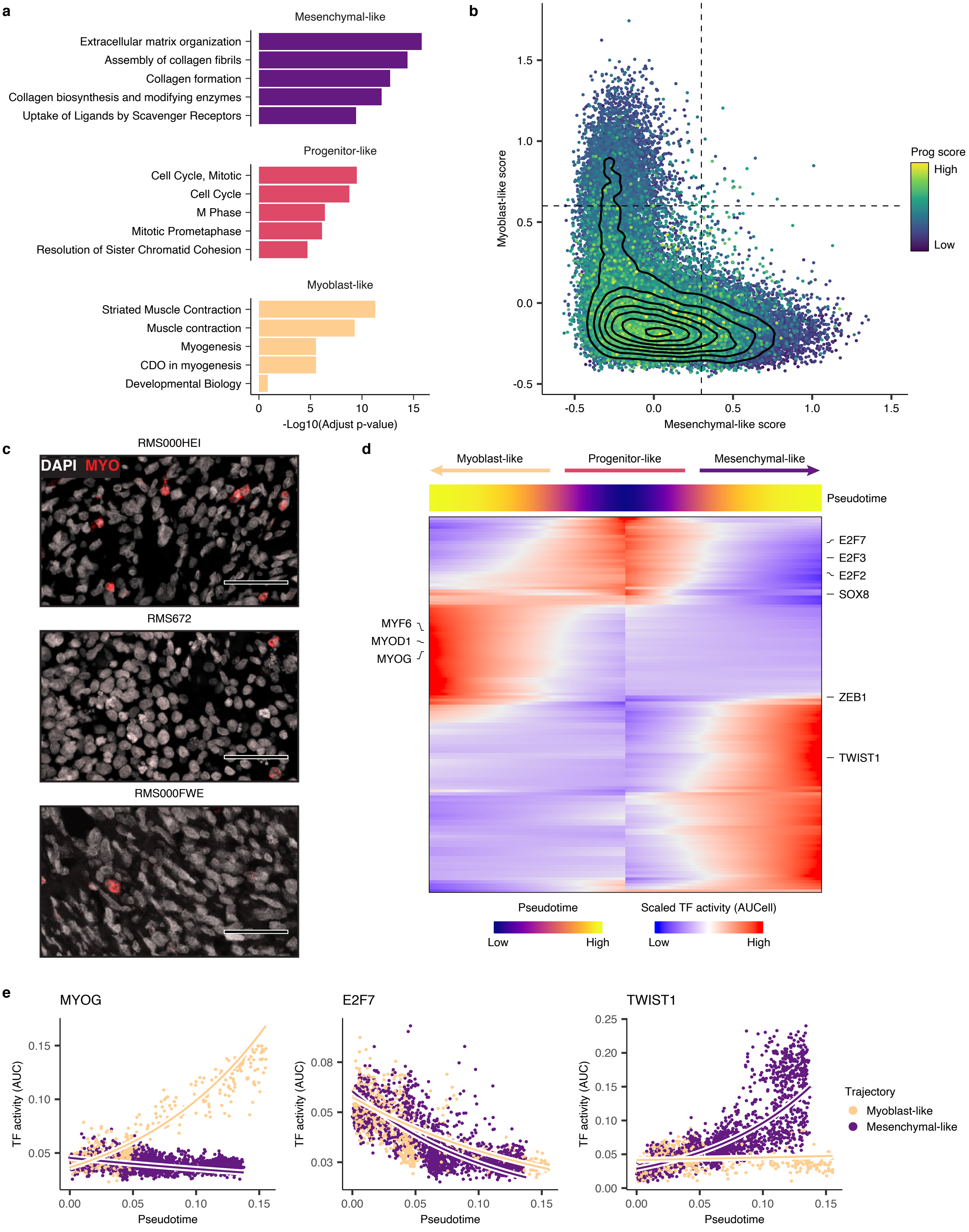

Supplementary Figure 5: Characterization of cell states in FN RMS tumours

a, Reactome pathway enrichment of the top 30 genes per FN meta-program. The top 5 terms per meta-program are labelled on the y-axis and the -Log10-transformed adjusted *p*-values (two-sided Fisher's exact test, Benjamini-Hochberg adjusted), are depicted by horizontal bars (coloured per meta-program). **b**, Scatter plot depicting the mesenchymal (x-axis), myogenic (y-axis) and progenitor-like (Prog, point colour) meta-program scores calculated in the dataset from Patel et. al[2] (malignant FN cells). Vertical and horizontal lines depict the discrete cell-state cut-offs used in Fig. 3b. Density contours are overlaid in black. **c**, Representative RNA-FISH images depicting the expression a myogenic cell-state marker gene in red (MYO = *TTN*) in FN tissue samples. DAPI counterstaining shown in grey. Scale bars equivalent to 50μm. **d,** Heatmap showing the TF activity (smoothed AUCell values, scaled per TF) as a function of pseudotime (x-axis, displayed in the top annotation track) along the myoblast-like and mesenchymal-like trajectories (indicated by top arrows). Selected TFs are highlighted in the plot margins. **e**, Scatter plots showing the relationship between pseudotime (x-axis) and activity (AUCell, y-axis) for selected TFs in FN RMS single cells (dots). Colours indicate which trajectory (myoblast-like or mesenchymal-like) each cell belongs to. Lines depict the smoothed activity per trajectory. Source data are provided as a Source Data file.

Supplementary Figure 6

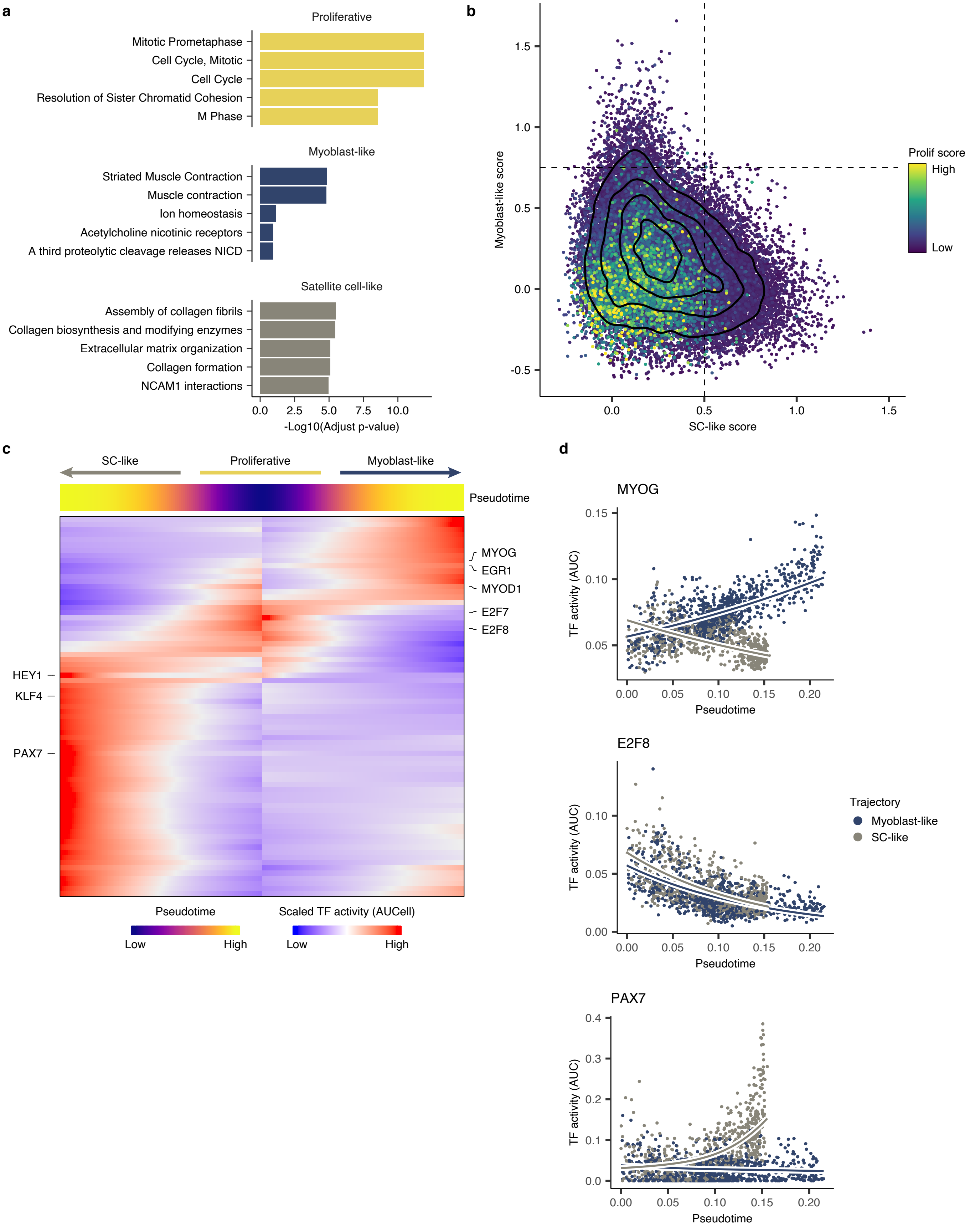

Supplementary Figure 6: Cell state characterization of FP RMS tumours

a, Reactome pathway enrichment of the top 30 genes per FP metaprogram. The top 5 terms per metaprogram are labelled on the y-axis and the -Log10-transformed adjusted *p*-values (two-sided Fisher's exact test, Benjamini-Hochberg adjusted) are depicted by horizontal bars (coloured per meta-program). **b**, Scatter plot depicting the satellite cell-like (SC-like, x-axis), myoblast-like (y-axis) and proliferative (Prolif, colour) meta-program scores calculated in the data from Patel et. al[2] (malignant FP cells). Vertical and horizontal lines depict the discrete cell-state cut-offs used in Fig. 4b. Density contours are overlaid in black. **c,** Heatmap showing the TF activity (smoothed AUCell values, scaled per TF) as a function of pseudotime (x-axis, displayed in the top annotation track) along the satellite cell-like (SC-like) and myoblast-like trajectories (indicated by top arrows). Selected TFs are highlighted in the plot margins. **d**, Scatter plots showing the relationship between pseudotime (x-axis) and activity (AUCell, y-axis) for selected TFs in FP RMS single cells (dots). Colours indicate which trajectory (SC-like or myoblast-like) each cell belongs to. Lines depict the smoothed activity per trajectory. Source data are provided as a Source Data file.

Supplementary Figure 7

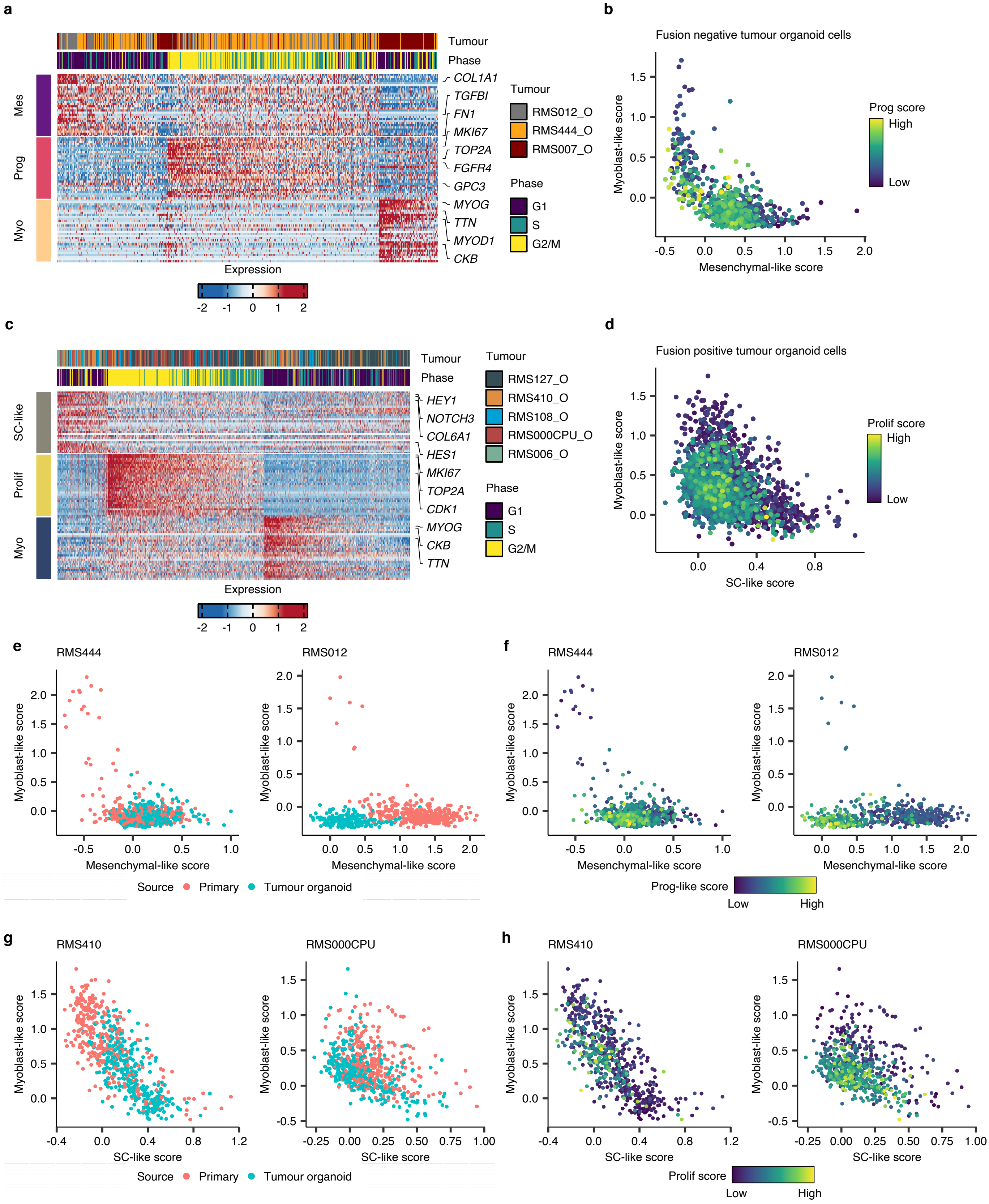

Supplementary Figure 7: Cell state heterogeneity in RMS tumour organoid models

a, Scaled expression of the top 30 genes per meta-program across all FN tumour organoid cells (Myo = Myoblast-like, Prog = Progenitor-like and Mes = Mesenchymal-like). The corresponding tumour organoid sample and inferred cell cycle phase of each cell are displayed in the top annotation track. Representative genes from each meta-program are labelled. **b**, Scatterplot depicting the mesenchymal-like (x-axis), myoblast-like (y-axis) and progenitor-like (Prog, point colour) meta-program scores for each FN tumour organoid cell. **c**, Scaled expression of the top 30 genes per meta-program across all FP tumour organoid cells (Myo = Myoblast-like, Prolif = Proliferative and SC-like = satellite cell-like). The corresponding tumour organoid sample and inferred cell cycle phase of each cell are displayed in the top annotation track. Representative genes from each meta-program are labelled. **d**, Scatterplot depicting the satellite cell-like (SC-like, x-axis), myoblast-like (y-axis) and proliferative (Prolif, point colour) meta-program scores for each FP tumour organoid cell. **e**, Scatterplots depicting mesenchymal-like (x-axis) and myoblast-like (y-axis) meta-program scores for paired FN primary and tumour organoid cells (indicated by point colour) derived from RMS444 (left panel) and RMS012 (right panel). **f**, Scatterplots depicting mesenchymal-like (x-axis), myoblast-like (y-axis) and progenitor-like (Prog-like, point colour) meta-program scores for paired FN primary and tumour organoid cells (indicated by point colour) derived from RMS444 (left panel) and RMS012 (right panel). **g**, Scatterplots depicting satellite cell-like (SC-like, x-axis) and myoblast-like (y-axis) meta-program scores for paired FP primary and tumour organoid cells (indicated by point colour) derived from RMS410 (left panel) and RMS000CPU (right panel). **h**, Scatterplots depicting satellite cell-like (SC-like, x-axis), myoblast-like (y-axis) and proliferative (Prolif, point colour) meta-program scores for paired FP primary and tumour organoid cells derived from RMS410 (left panel) and RMS000CPU (right panel). Source data are provided as a Source Data file.

Supplementary Figure 8

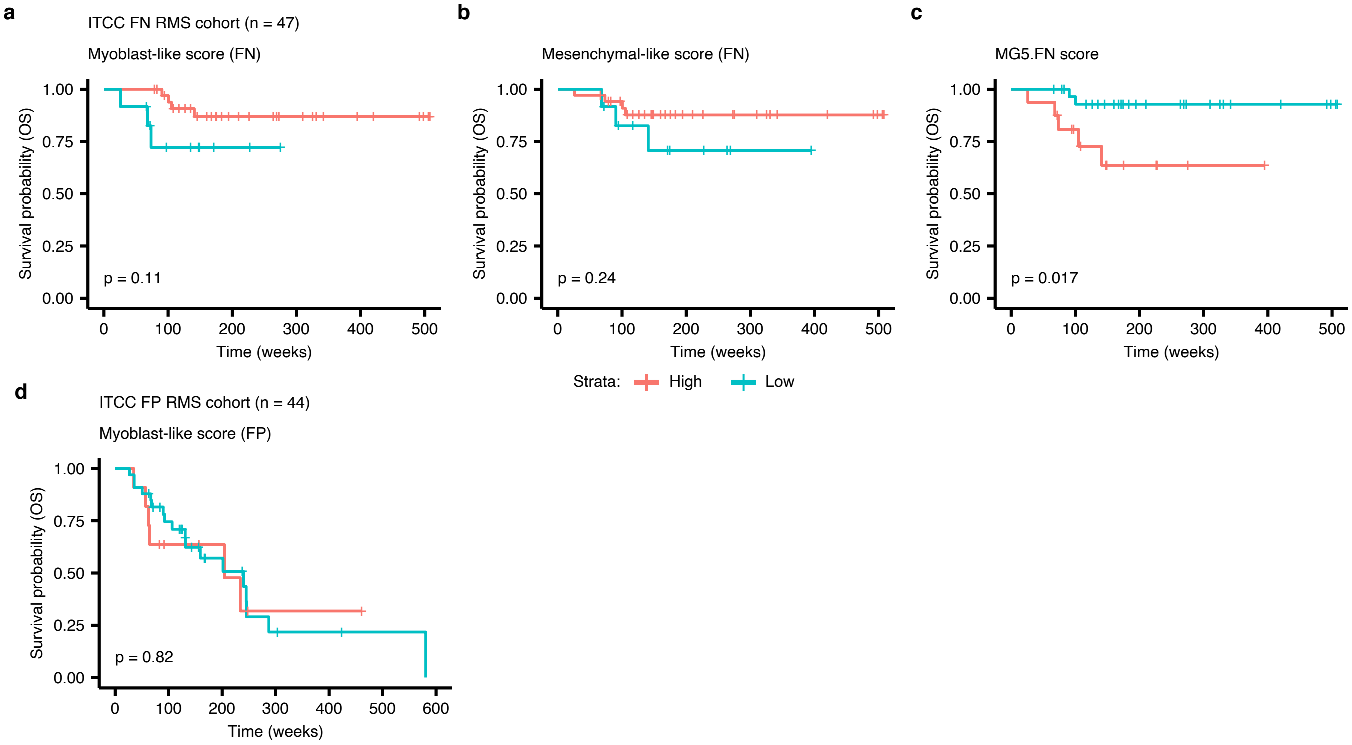

Supplementary Figure 8: Analysis of the relationship between transcriptional program scores and patient outcomes

Kaplan-Meier plots showing the overall survival (OS) probabilities of (**a, b, c**) FN (n = 47) or (**d**) FP (n = 44) ITCC patients divided into high (pink strata) or low (blue strata) groups based on their cell state or MG5.FN scores (stated in the title of each plot panel). Log-rank test was used to calculate p values between high- and low- scoring groups. Source data are provided as a Source Data file.

Supplementary Table 1	
**M1**	**M2**
IL23	IL4R
TNF	CCL4
CXCL9	CCL13
CXCL10	CCL20
CXCL11	CCL17
CD86	CCL18
IL1A	CCL22
IL1B	CCL24
IL6	LYVE1
CCL5	VEGFA
IRF5	VEGFB
IRF1	VEGFC
CD40	VEGFD
IDO1	EGF
KYNU	CTSA
CCR7	CTSB
	CTSC
	CTSD
	TGFB1
	TGFB2
	TGFB3
	MMP14
	MMP19
	MMP9
	CLEC7A
	WNT7B
	FASL
	TNFSF12
	TNFSF8
	CD276
	VTCN1
	MSR1
	FN1
	IRF4

Supplementary Table 1: Genes used to define polarization states of macrophages

All genes used for calculating “M1” and “M2” scores for macrophages, as derived from Cheng et al.[3]

Supplementary Table 2		
**Progenitor-like**	**Myoblast-like**	**Mesenchymal-like**
NNAT	ACTC1	COL6A1
CENPF	MYOG	COL6A3
TMPO	TNNT2	DCN
NCAPD2	TNNI1	COL1A1
MKI67	OLFML2B	COL6A2
CDK1	TTN	POSTN
HMGB2	ACTN2	COL1A2
STMN1	FNDC5	COL5A2
NUSAP1	SEPTIN4	COL3A1
H2AFZ	MYOD1	LRP1
TOP2A	RASSF4	FTL
TUBB	MYL4	PXDN
FGFR4	BIN1	NGFR
ANLN	CHRND	KCTD12
FLNC	MEF2C	TIMP1
BIRC5	MYH3	COL4A2
PRC1	RYR1	SPARC
AURKB	ARPP21	MMP2
H2AFV	ELFN1	EMP1
HMGB1	MYL1	TGFBI
RRM2	CASS4	CAVIN1
TMSB15A	CKB	LGALS1
GPC3	MTSS2	CREB3L1
CHRNA1	SNTB1	B2M
HMGA2	C1orf105	ITM2C
HIST1H4C	B4GALNT3	RRBP1
TPX2	COBL	LOXL2
CENPU	MYLPF	TMSB10
KIF11	ADGRB1	NEAT1
BCAT1	CDH15	FN1

Supplementary Table 2: Top 30 FN meta-program genes

Genes used to define each meta-program in FN RMS cells

**Supplementary Table 3**		
**Proliferative**	**Myoblast-like**	**SC-like**
MKI67	RYR1	COL5A1
TOP2A	ACTC1	HEY1
NUF2	RASSF4	NOTCH3
TPX2	TNNT2	COL6A2
CENPF	MYOG	COL6A1
HMGB2	CKB	PEG10
NUSAP1	NES	COL4A2
AURKB	SOX8	PARM1
NCAPD2	MIR1-1HG-AS1	IFITM3
CDK1	MYL4	AHNAK
TROAP	TTN	CALD1
ASPM	LMF1	COL4A1
TACC3	FNDC5	ITIH5
KIF11	SIRT2	GBP2
CKAP2	EMC10	PDGFA
KNL1	FLNC	EPAS1
KIF14	PDLIM4	ZBTB20
GTSE1	HSPB1	JUN
MXD3	TNNI1	EEF1A1
ARHGAP11A	PDLIM3	GPC3
HJURP	PLS3	PRRX1
BIRC5	ZBTB18	NFIA
SMC4	DMPK	B2M
BUB1B	SELENOW	SLC27A6
CENPE	SEMA6A	HES1
RACGAP1	CHRND	DLC1
RRM2	INPPL1	MALAT1
ANLN	BLCAP	SDC3
SKA3	PSEN2	GPC6
CDCA4	AC132217.2	OBSL1

Supplementary Table 3: Top 30 FP meta-program genes

Genes used to define each meta-program in FP RMS cells

Supplementary references

1. Meister, M. T. *et al.* Mesenchymal tumor organoid models recapitulate rhabdomyosarcoma subtypes. *EMBO Mol Med* **14**, (2022).
2. Patel, A. G. *et al.* The myogenesis program drives clonal selection and drug resistance in rhabdomyosarcoma. *Dev Cell* **57**, (2022).
3. Cheng, S. *et al.* A pan-cancer single-cell transcriptional atlas of tumor infiltrating myeloid cells. *Cell* **184**, (2021).
