# Supplementary material for: Single-cell transcriptomics reveals immune suppression and cell states predictive of patient outcomes in rhabdomyosarcoma
Source: Nat Commun. 2023 May 27;14:3074. doi: 10.1038/s41467-023-38886-8 (PMC10224926; doi:10.1038/s41467-023-38886-8)
Supplement: Supplementary file 2 — Description of Additional Supplementary Files [file 41467_2023_38886_MOESM2_ESM.pdf]

Description of Additional Supplementary Files

File Name: Supplementary Data 1
Description: Detailed overview of RMS patient and sample characteristics

File Name: Supplementary Data 2
Description: FN meta-program gene weights

File Name: Supplementary Data 3
Description: FP meta-program gene weights
