# Supplementary material for: Single-cell transcriptomics reveals immune suppression and cell states predictive of patient outcomes in rhabdomyosarcoma
Source: Nat Commun. 2023 May 27;14:3074. doi: 10.1038/s41467-023-38886-8 (PMC10224926; doi:10.1038/s41467-023-38886-8)
Supplement: Supplementary file 6 — Reporting Summary [file 41467_2023_38886_MOESM6_ESM.pdf]

Corresponding author(s): Jarno Drost
Thanasis Margaritis

Last updated by author(s): May 19, 2023

Reporting Summary

Nature Portfolio wishes to improve the reproducibility of the work that we publish. This form provides structure for consistency and transparency in reporting. For further information on Nature Portfolio policies, see our Editorial Policies and the Editorial Policy Checklist.

Statistics

For all statistical analyses, confirm that the following items are present in the figure legend, table legend, main text, or Methods section.

n/a	Confirmed	
☐	☒	The exact sample size (*n*) for each experimental group/condition, given as a discrete number and unit of measurement
☐	☒	A statement on whether measurements were taken from distinct samples or whether the same sample was measured repeatedly
☐	☒	The statistical test(s) used AND whether they are one- or two-sided<br>*Only common tests should be described solely by name; describe more complex techniques in the Methods section.*
☐	☒	A description of all covariates tested
☐	☒	A description of any assumptions or corrections, such as tests of normality and adjustment for multiple comparisons
☐	☒	A full description of the statistical parameters including central tendency (e.g. means) or other basic estimates (e.g. regression coefficient) AND variation (e.g. standard deviation) or associated estimates of uncertainty (e.g. confidence intervals)
☐	☒	For null hypothesis testing, the test statistic (e.g. *F*, *t*, *r*) with confidence intervals, effect sizes, degrees of freedom and *P* value noted<br>*Give P values as exact values whenever suitable.*
☒	☐	For Bayesian analysis, information on the choice of priors and Markov chain Monte Carlo settings
☐	☒	For hierarchical and complex designs, identification of the appropriate level for tests and full reporting of outcomes
☐	☒	Estimates of effect sizes (e.g. Cohen's *d*, Pearson's *r*), indicating how they were calculated

Our web collection on statistics for biologists contains articles on many of the points above.

Software and code

Policy information about availability of computer code

Data collection

LAS X (Leica, v5.1.0) used for microscopy imaging acquisition
SONY SH800S system software (v2.1)

Data analysis

R (v4.1.0, https://www.r-project.org/)
Python (v3.7, https://www.python.org/)

zUMIs (v2.5.6b, https://github.com/sdparekh/zUMIs)
samtools (v1.9, https://github.com/samtools/samtools)
STAR (v2.6.0c, https://github.com/alexdobin/STAR)
Seurat (v4.1.0, https://github.com/satijalab/seurat)
SingleR (v1.6.1, https://github.com/LTLA/SingleR)
InferCNV (v1.8.0, https://github.com/broadinstitute/infercnv)
celldex (v1.2.0, https://github.com/LTLA/celldex)
msigdbr (v7.4.1, https://github.com/igordot/msigdbr)
fgsea (v1.20.0, https://github.com/ctlab/fgsea)
CellChat (v1.0.0, https://github.com/sqjin/CellChat)
glmnet (v4.1-3, https://github.com/cran/glmnet)
NMF (v0.23, https://github.com/cran/NMF)
enrichR (v3.0, https://github.com/wjawaid/enrichR)
destiny (v3.1.1, https://github.com/theislab/destiny)
Slingshot (v2.0.0, https://github.com/kstreet13/slingshot)

survival (v3.2-11, https://github.com/therneau/survival)
scVelo (v0.2.2, https://github.com/theislab/scvelo)
pySCENIC (v0.11.2, https://github.com/aertslab/pySCENIC)
tradeSeq (v1.10.0, https://github.com/statOmics/tradeSeq)

FIJI (v2.0.0-rc-69/1.52i, https://fiji.sc/)

FlowJo v10.8 (BD Life Sciences)

For manuscripts utilizing custom algorithms or software that are central to the research but not yet described in published literature, software must be made available to editors and reviewers. We strongly encourage code deposition in a community repository (e.g. GitHub). See the Nature Portfolio guidelines for submitting code & software for further information.

Data

Policy information about availability of data

All manuscripts must include a data availability statement. This statement should provide the following information, where applicable:

- Accession codes, unique identifiers, or web links for publicly available datasets
- A description of any restrictions on data availability
- For clinical datasets or third party data, please ensure that the statement adheres to our policy

The raw sequencing data generated in this study have been deposited in the European genome-phenome archive. The accession number for the single-cell RNA-sequencing data is EGAD00001009385 (https://ega-archive.org/datasets/EGAD00001009385; "Single-cell mRNA-sequencing to generate a transcriptomic atlas of RMS"). To protect patient privacy, as required by law, access to the sequencing data deposited in the EGA is controlled by the Data Access Committee (DAC) of the Princess Maxima Center. All researchers can obtain access by submitting a project proposal to the DAC (biobank-2 [at] prinsesmaximacentrum [dot] nl). Requests will be handled within approximately 2 weeks. The DAC will also determine the length of permitted access. The publicly available whole genome sequencing data generated by Meister et al and used in Supplementary Fig. 2 can be accessed with the identifier EGAD00001008466 (https://ega-archive.org/datasets/EGAD00001008466; “WGS soft tissue sarcoma tumoroid biobank”)16. The publicly available bulk RNA-sequencing data generated by Meister et al and used in Supplementary Fig. 1d can be accessed with the identifier EGAD00001008467 (https://ega-archive.org/datasets/EGAD00001008467; “RNA-Seq soft tissue sarcoma tumoroid biobank”).

The publicly available single-nucleus RNA-sequencing data of RMS tumours generated by Patel et al and used in this study are deposited in the single-cell paediatric cancer atlas portal under accession number SCPCP000005 (https://scpca.alexslemonade.org/projects/SCPCP000005) The publicly available single-cell RNA-sequencing data of normal myogenic differentiation used in this study are deposited in the GEO repository under accession number GSE147457 (https://www.ncbi.nlm.nih.gov/geo/query/acc.cgi?acc=GSE147457) and were accessed from https://skeletal-muscle.cells.ucsc.edu. The publicly available ITCC RMS microarray dataset is deposited in the gene expression omnibus (GEO) under accession number GSE92689 (https://www.ncbi.nlm.nih.gov/geo/query/acc.cgi?acc=GSE92689) and were accessed from the R2 Genomics Analysis and Visualization Platform (http://r2.amc.nl).

The GRCh38v2020-A reference genome used to map the single-cell RNA-seq data was downloaded from the 10X genomics website (https://support.10xgenomics.com/single-cell-gene-expression/software/release-notes/build). The publicly available Human Primary Cell Atlas data, Novershtern Hematopoietic data and Monaco Immune data were accessed using the celldex R package (v1.2.0, https://github.com/LTLA/celldex). The publicly available CellPhoneDB ligand-receptor interaction data was accessed from https://www.cellphonedb.org (RRID: SCR_017054). The publicly available auxiliary input databases for the SCENIC analysis were accessed from https://resources.aertslab.org/cistarget/. The processed data used in and generated by this study, including compiled count tables and processed R objects, have been made publicly available via Zenodo (https://doi.org/10.5281/zenodo.7928694). The remaining data are available within the Article, Supplementary Information or Source Data file. Source data are provided with this paper.

Human research participants

Policy information about studies involving human research participants and Sex and Gender in Research.

Reporting on sex and gender	Patient sex information was collected and mentioned in Supplementary Data 1, Figure 1b and Supplementary Figure 1b. However, this information was not a criterion for study design or data interpretation.
Population characteristics	Material from patients diagnosed with RMS and treated at the Princess Maxima Center were included in this study irrespective of age, sex or any other characteristics.
Recruitment	Tumour samples of RMS were obtained via an established sample acquisition route as part of the biobank initiative of the Princess Máxima Center for Pediatric Oncology, Utrecht, Netherlands (remaining tumour samples). All patients and/or their legal representatives signed informed consent to have tumour samples taken for biobank usage. Experiments conformed to the principles set out in the WMA Declaration of Helsinki and the Department of Health and Human Services Belmont Report.
Ethics oversight	Ethics approval was granted for the biobanking initiative by the Medical Research Ethics Committee (METC) of the University Medical Center Utrecht, and the Maxima biobank committee granted approval for the present project.

Note that full information on the approval of the study protocol must also be provided in the manuscript.

Field-specific reporting

Please select the one below that is the best fit for your research. If you are not sure, read the appropriate sections before making your selection.

☒ Life sciences ☐ Behavioural & social sciences ☐ Ecological, evolutionary & environmental sciences

For a reference copy of the document with all sections, see nature.com/documents/nr-reporting-summary-flat.pdf

Life sciences study design

All studies must disclose on these points even when the disclosure is negative.

Sample size

The number of samples for which single-cell RNA-sequencing was performed was not predetermined. All samples for which material was or became available during the course of the experiment were sequenced. Immunohistochemical, immunofluoresence and RNAscope experiments were performed on all tumours within the single-cell RNA-sequencing cohort for which biobanked tissue (FFPE) was available.

Data exclusions

Cells which could not be defined as malignant or normal were excluded from the final dataset. Samples with fewer than 30 malignant single-cells were excluded from the NMF analysis. Additionally, RMS000FAN was excluded from the NMF analysis as it did not pass quality control assessment and RMS000DJE was excluded from NMF analysis due to atypical (sclerosing) histological classification.

Replication

Individual patients served as biological replicates when performing molecular subtype specific analyses. The relationship(s) between NMF program scores was replicated using an independent RMS single-nucleus RNA-sequencing dataset (Patel et al). Immunohistochemical, immunofluorescence and RNAscope stainings were carried out once per patient due to limited material availability. When interpreting imaging results, at least 3 randomly chosen fields were surveyed. All attempts at replication were successful.

Randomization

Not relevant, as no allocation to experimental groups was performed.

Blinding

Blinding was not relevant to this study, as data collection and analysis required knowledge of the sample being collected and/or analysed

Reporting for specific materials, systems and methods

We require information from authors about some types of materials, experimental systems and methods used in many studies. Here, indicate whether each material, system or method listed is relevant to your study. If you are not sure if a list item applies to your research, read the appropriate section before selecting a response.

Materials & experimental systems

n/a	Involved in the study	
☐	☒	Antibodies
☐	☒	Eukaryotic cell lines
☒	☐	Palaeontology and archaeology
☒	☐	Animals and other organisms
☒	☐	Clinical data
☒	☐	Dual use research of concern

Methods

n/a	Involved in the study	
☒	☐	ChIP-seq
☐	☒	Flow cytometry
☒	☐	MRI-based neuroimaging

Antibodies

Antibodies used

anti-NECTIN3 (R&D systems, AF3064, 1:200 dilution of a 0.2µg/µl solution in PBS)
anti-TIGIT clone E5Y1W (Cell Signaling, #99567, 1:500 dilution)
anti-CD206/MRC1 clone E2L9N (Cell Signaling, #91992, 1:200 dilution)
anti-Goat Alexa 647 (Abcam, ab150131, diluted 1:1000)
anti-Rabbit Alexa 568 (Abcam, ab175470, diluted 1:1000)
anti-CD3 clone LN10 (Leica, PA0533)
anti-CD8 clone 4B11 (Leica, PA0183)
anti-CD68 clone 514H12 (Leica, PA0273)
anti-CD163 clone EPR19518 (Abcam, ab182422, diluted 1:200)

Validation

All antibodies used in this study were validated by their respective manufacturer. Description of the validations and references to other studies making use of them are available on the manufacturer's webpages:
https://www.rndsystems.com/products/human-nectin-3-antibody_af3064
https://www.cellsignal.com/products/primary-antibodies/tigit-e5y1w-xp-rabbit-mab/99567
https://www.cellsignal.com/products/primary-antibodies/cd206-mrc1-e2l9n-rabbit-mab/91992
https://www.abcam.com/donkey-goat-igg-hl-alexa-fluor-647-ab150131.html
https://www.abcam.com/donkey-rabbit-igg-hl-alexa-fluor-568-ab175470.html
https://shop.leicabiosystems.com/us/ihc-ish/ihc-primary-antibodies/pid-cd3

https://shop.leicabiosystems.com/us/ihc-ish/ihc-primary-antibodies/pid-cd8
https://shop.leicabiosystems.com/us/ihc-ish/ihc-primary-antibodies/pid-cd68
https://www.abcam.com/products/primary-antibodies/cd163-antibody-epr19518-ab182422.html

Eukaryotic cell lines

Policy information about cell lines and Sex and Gender in Research

Cell line source(s)	Patient-derived RMS tumour organoids (Establishment described in M Meister et al., 2022. EMBO Molecular Medicine):<br>RMS006_O - Male<br>RMS123_O - Male<br>RMS000CPU_O - Male<br>RMS108_O - Female<br>RMS410_O - Male<br>RMS444_O - Male<br>RMS012_O - Male<br>RMS007_O - Male
Authentication	Cell lines were authenticated through compared with the tumour they were derived from, as described in M Meister et al., 2022. EMBO Molecular Medicine
Mycoplasma contamination	All lines tested negative for mycoplasma contamination
Commonly misidentified lines<br>(See ICLAC register)	None used

Flow Cytometry

Plots

Confirm that:

☒ The axis labels state the marker and fluorochrome used (e.g. CD4-FITC).

☒ The axis scales are clearly visible. Include numbers along axes only for bottom left plot of group (a 'group' is an analysis of identical markers).

☒ All plots are contour plots with outliers or pseudocolor plots.

☒ A numerical value for number of cells or percentage (with statistics) is provided.

Methodology

Sample preparation	SORT-seq:<br>Viably frozen primary tumour samples were rapidly thawed in a water bath, minced using a scalpel and then transferred to a tube containing 4.5 ml of BM1* medium (Advanced DMEM/F12 [Gibco, cat no. 12634010] supplemented with 1% Glutamax [Gibco, cat no. 35050061], 1% Penicillin/Streptomycin [Gibco, cat no. 15140122], 2% B27 minus vitamin A [Gibco, cat no. 12587010], 1% N2 [Gibco, cat no. 17502048], 0.25% N-acetylcysteine [500 mM, Sigma, cat no. A9165], 1% MEM non-essential amino acids [Gibco, cat no. 11140035], 1% sodium pyruvate [100 mM, Gibco, cat no. 11360070], 0.01% heparin [5,000 U/ml, Sigma, cat no. H3149-10KU], 1% hEGF [2 µg/ml, Peprotech, cat no. AF-100-15], 0.1% hFGF-basic [40 µg/ml, Peprotech, cat no. 100-18B], 0.02% hIGF1 [100 µg/ml, Peprotech, cat no. 100-11], 0.01% Rho kinase inhibitor [Y-27632, 100 mM, AbMole Bioscience, cat no. M1817] and 0.1% A83-01 [5 mM, Tocris Bioscience, cat no. 2939]). To this, 0.5 ml of Collagenase D (Roche, #11088866001, 1:10 dilution) and DNAseI (Stemcell #07900, stock diluted 1:40 in PBS, further 1:100 diluted in the BM1* mixture) were added, and samples were allowed to dissociate in a shaker set to 250 rpm for 30 minutes at 37°C. Following digestion, samples were passed through a 70 µm strainer which was subsequently flushed with an additional 5ml of BM1* (supplemented with DNAseI) to increase the yield. Samples were then washed twice with 5ml of washing medium (Advanced DMEM/F12 supplemented with 1% Glutamax, 1% Penicilin/Streptomycin and 1% HEPES [1M, Gibco, cat no. 15630049]), centrifuging at 300g for 5 minutes (at 4°C) in between steps. After the final washing step samples were resuspended in BM1* (supplemented with DNAseI) to a final concentration of < 1 x 106 cells per ml. Viably frozen tumour organoid samples were rapidly thawed in a water bath and immediately resuspended in BM1* (supplemented with DNAseI) to a final concentration of < 1 x 106 cells per ml. Prior to sorting, 4′,6-diamidino-2-phenylindole (DAPI, Sigma-Aldrich, #D9542) and DRAQ5 (Thermo Fisher, #65-0880-92) were added to single-cell suspensions up to final concentrations of 1µM and 5µM, respectively.
Instrument	SORT-seq: SONY SH800S
Software	SORT-seq: SONY SH800S system software (v2.1)
Cell population abundance	SORT-seq: Viable single cells (DRAQ5+/DAPI-) were sorted in 384-well plates.
Gating strategy	SORT-seq: DRAQ5+ cells were selected by assessing the distribution of DRAQ5-A on a per sample basis. From this population, DAPI- cells were retained by assessing the distribution of DAPI-A on a per sample basis. We retained singlets by assessing the relationship between FSC-A vs FSC-H and SSC-A vs SSC-H per sample.

☒ Tick this box to confirm that a figure exemplifying the gating strategy is provided in the Supplementary Information.
